# Molecular mechanisms of sulforaphane in Alzheimer’s disease: insights from an in-silico study

**DOI:** 10.1007/s40203-024-00267-4

**Published:** 2024-11-01

**Authors:** Giang Huong Vu, Hai Duc Nguyen

**Affiliations:** 1 Department of Public Heath, Hong Bang Health Center, Hai Phong, Vietnam; 2 Department of Pharmacy, College of Pharmacy and Research Institute of Life and Pharmaceutical Sciences, Sunchon National University, Suncheon, 57922 Republic of Korea

**Keywords:** Sulforaphane, Alzheimer’s disease, Molecular mechanisms, Post-translational modification, In silico

## Abstract

This study was to identify the molecular pathways that may explain sulforaphane’s Alzheimer’s disease (AD) benefits using multiple advanced in silico approaches. We found that sulforaphane regulates 45 targets, including TNF, INS, and BCL2. Therefore, it may help treat AD by reducing neuroinflammation, insulin resistance, and apoptosis. The important relationships were co-expression and pathways. 45 targets were linked to the midbrain, metabolite interconversion enzymes, 14q23.3 and 1q31.1 chromosomes, and modified residues. “Amyloid precursor protein catabolic process”, “regulation of apoptotic signaling pathway”, and “positive regulation of nitric oxide biosynthetic process” were the main pathways, while NFKB1, SP1, RELA, hsa-miR-17-5p, hsa-miR-16-5p, and hsa-miR-26b-5p were transcription factors and miRNAs implicated in sulforaphane In AD treatment, miRNA sponges, dexibuprofen, and sulforaphane may be effective. Furthermore, its unique physicochemical, pharmacokinetic, and biological qualities make sulforaphane an effective AD treatment, including efficient gastrointestinal absorption, drug-like properties, absence of CYP450 enzyme inhibition, not being a substrate for P-glycoprotein, ability to cross the blood–brain barrier, glutathione S-transferase substrate, immunostimulant effects, and antagonistic neurotransmitter effects. Sulforaphane is a promising compound for AD management. Further work is needed to elucidate its therapeutic effects based on our findings, including genes, miRNAs, molecular pathways, and transcription factors.

**Supplementary Information:**

The online version contains supplementary material available at 10.1007/s40203-024-00267-4.

## Introduction

Sulforaphane, also known as 1-isothiocyanato-4-methylsulfonylbutane, is a highly effective bioactive compound derived from various cruciferous vegetables, including cabbage, collard greens, cauliflower, broccoli, kale, mustard greens, brussels sprouts, bok choy, and watercress (Palliyaguru et al. 2018). Sulforaphane exhibits robust antioxidant, anti-inflammatory, and antiapoptotic properties, rendering it a promising candidate for therapeutic use in many neurodegenerative disorders (de Figueiredo et al. 2015; Schepici et al. 2020). There is an increasing body of research suggesting that sulforaphane has a protective effect against Alzheimer’s disease (AD) through mitigating neuroinflammation, tau hyperphosphorylation, amyloid-β accumulation, and oxidative stress in vivo and in vitro models (Table 1). While the therapeutic effects of sulforaphane have been confirmed in vivo or in vitro, only one clinical trial study is being conducted on its effects on AD (Second Affiliated Hospital 2020). Certain pathways related to AD and sulforaphane are not yet fully understood. Therefore, the identification of precise molecular processes might be regarded as a valuable therapeutic objective in the development of novel methods aimed at mitigating the advancement of AD.

**Table 1:** The association between sulforaphane and Alzheimer’s disease by the literature review approach

Study design	Sulforaphane doses	Control groups	Exposure duration	Statistical significance	Main findings	Possible outcomes	References
In vivo models							
3 × Tg-AD mice	Daily 10–50 mg/kg p.o. (for 2 months, 6 days/week)	Non-transgenic mice treated with phosphate-buffered saline (PBS) vehicle	Sulforaphane (SFN) or vehicle was administered by oral gavage (p.o.) 6 days a week for 8 weeks	ANOVA followed by Fisher’s least significant difference tests, p-value < 0.05	↓Aβ level ↓Tau and p-tau level ↑Heat shock protein 70 and cochaperone C terminus of Hsc70-interacting protein	↓Cognitive deficit	Lee et al. (2018)
PS1V97L mice	Daily 5 mg/kg i.p. (for 4 months)	Six-month-old PS1V97L Tg105 mice (n = 6) were treated with corn oil	5 mg/kg 106 SFN in corn oil intraperitoneally 107 every day for 4 months until 10 months of age	Student’s t-test or the Mann–Whitney Utest, p-value < 0.05	↓Aβ oligomers ↓Tau hyperphosphorylation ↓Serum beta-secretase 1 (BACE1) and presenilin-1 ↓IL-1β and TNF-α ↑Glutathione and malondialdehyde	↓Cognitive deficit	Hou et al. (2018)
3 × Tg-AD mice	10 or 50 mg/kg p.o. (6 days/week for 2 months)	Non-transgenic mice treated with vehicle (PBS, 100 μl/day; n = 10)	Mice treated with 10 mg/kg/day sulforaphane (100 μl/day; n = 10), or 50 mg/kg/day sulforaphane (100 μl/day; n = 10)	Kruskal–Wallis tests, p-value < 0.05	↑Synaptophysin, microtubule-associated protein 2, and Postsynaptic density protein 95 ↑TrkB signaling pathway ↑Brain derived neurotrophic factor	↓Alzheimer’s disease	Kim et al. (2017)
AD-like rat caused by Aβ_42_	Daily 5 mg/kg i.p. (for 7 days)	Rats were treated daily with corn oil	SFN in corn oil was injected intraperitoneally at 5 mg/kg per day for 7 days	ANOVA (Bonferroni), p-value < 0.05	↓IL-1β and TNF-α ↑Glutathione and malondialdehyde	↓Memory impairment and depressive-like behavior	Wang et al. (2020)
3 × Tg-AD mice and 5 × FAD mice	10 mg/kg i.p (for 2 months, every other day)	5xFAD, 3xTg-AD, 12-month-old C57BL/6 J (male) and Nrf2 − / − mice (male) were injected intraperitoneally with 5% DMSO, 95% PBS	Mice were injected intraperitoneally with 5% DMSO, 95% PBS or 5 or 10 mg/kg sulforaphane every other day for 2 months	ANOVA with Tukey’s, or two-way ANOVA, p-value < 0.05	↓Aβ1-40 and Aβ1-42 ↓p-tau level ↓BACE1 mRNA, protein, and BACE1-antisense transcript ↑HO-1 mRNA and protein ↑NAD(P)H Quinone dehydrogenase 1 transcript and protein	↓Cognitive deficit	Bahn et al. (2019)
APP/PS1 mice	Daily 25 mg/kg p.o. (for 5 months)	Wild-type (WT) mice (n = 10)	Mice in the WT + SFN and AD + SFN groups were treated with 25 mg/kg SFN (dissolved in distilled water) or distilled water by a single oral gavage daily up to 5 months	ANOVA followed by Fisher’s least significant difference tests, p-value < 0.05	↓Aβ plaques ↓Histone deacetylase 1 and 3 (HDAC1 and 3) ↑Neurotrophin receptor p75 ↑Histone acetylation (acetylated histone 3 lysine 9 (Ace-H3K9) and acetylated histone 4 lysine 12 (Ace-H4K12))	↓Cognitive deficit	Zhang et al. (2017)
In vitro models							
Aβ_25-35_ treatment in mouse microglial cell line	1 μM co-treatment with Aβ_25–35_ (for 3-24 h)	Primary microglia were treated with different concentrations of either Aβ	12 or 24 h	Ordinary-one-way ANOVA test, p-value < 0.05	↓Reactive oxygen species ↓Cytotoxic autophagy ↓NLRP3/caspase-1 inflammasomes ↓ Neuronal damage ↓M1 polarization	↓Alzheimer’s disease	Yang et al. (2023)
Aβ_25-35_ treatment in human neuroblastoma SH-SY5Y cells	1 μM co-treatment with Aβ_25–35_ (for 24-72 h)	SH-SY5Y cells were incubated with 25 μM Aβ 25–35 in the absence of 10 μL/mL of juice A or juice B	24, 48, and 72 h	Unpaired Student’s t-test, p-value < 0.05	↑Glutathione and heme oxygenase 1 (HO-1) ↑Thioredoxin and its receptor expression ↑NAD(P)H:quinone oxidoreductase 1 (NQO1) activity ↑Nuclear factor erythroid 2–related factor 2 (Nrf2) ↓Cell death ↑70 kilodalton heat shock proteins	↓Alzheimer’s disease	Masci et al. (2015)
Aβ_25-35_ treatment in human neuroblastoma SH-SY5Y cells	Pre-treatment 2 μM for 3 h and then Aβ_25–35_	Cells were incubated without SFN (final concentration 2 μM)	3-h	ANOVA followed by Fisher’s least significant difference tests, p-value < 0.05	↓Cell death ↓HDAC1 and 3 ↑Neurotrophin receptor p75 ↑Ace-H3K9 and Ace-H4K12	↓Alzheimer’s disease	Zhang et al. (2017)
Aβ_25-35_ treatment in human neuroblastoma SH-SY5Y cells	Pre-treatment 1–5 μM for 30 min and then Aβ_25–35_	Cells were incubated with Aβ 25–35 (15 μM) for 24 h without pretreatment of SFN (1, 2, and 5 μM)	30-min	ANOVA followed by Tukey’s test, p-value < 0.05	↓Reactive oxygen species ↑NQO1, HO-1 and γ-glutamylcysteine ligase ↑Nrf2 ↓Bax/Bcl-2 and cell death ↓c-Jun N-terminal kinases	↓Alzheimer’s disease	Lee et al. (2013)
Aβ1-42 treatment in mouse neuroblastoma N2A cells	Pre-treatment 2.5 μM for 18 h and then Aβ_1–42_	Neuro 2A cells were pre-incubated without 2.5 μM SFN and the Aβ1-42 peptide (10 and 50 μM)	18-h	One-way ANOVA followed by the Newman-Keuls, p-value < 0.05	↓Cell death - Depending on proteasome activity	↓Alzheimer’s disease	Park et al. (2009)
Aβ1-42 treatment in mouse neuroblastoma N1E-115	Pre-treatment 5 μM for 18 h and then Aβ_1–42_	Neuro 2A cells were pre-incubated without 2.5 μM SFN and the Aβ1-42 peptide (10 and 50 μM)	18-h	One-way ANOVA followed by the Newman-Keuls, p-value	↓Cell death	↓Alzheimer’s disease	Park et al. (2009)
Aβ1-42 treatment in mouse EOC-20 microglial cells	5 μM co-treatment with Aβ1–42 (for 24 h)	Different concentrations of Aβo (100, 500, and 1000 ng/mL) in presence or absence of SFN (5 µM)	24-h	One-way ANOVA followed by the Newman-Keuls, p-value	↑Phagocytic activity ↑Formyl Peptide Receptor 2	↓Alzheimer’s disease	Chilakala et al. (2020)
Murine neuroblastoma N2a expression of APPs	1.25–2.5 μM (for 48 h)	Cells were treated with 0.1% DMSO, sulforaphane (1.25 and 2.5 μM), or 5 μM of 5-Aza-dC	48-h	ANOVA followed by Dunnett's or Tukey’s test, p-value	↓Aβ1-40 and Aβ1-42 ↓Reactive oxygen species and malondialdehyde ↑Superoxide dismutase activity ↑Nrf2 expression	↓Alzheimer’s disease	Zhao et al. (2018)
3 × Tg-AD mice primary cortical neurons	10 μM (for 6 h)	Vehicle treatment	6-h	ANOVA followed by Fisher’s least significant difference tests, p-value	↓Aβ and tau ↑C-terminus of HSP70-interacting protein	↓Alzheimer’s disease	Lee et al. (2018)
Treatment of human microglia-like THP-1 cells with Aβ1–42	Pre-treatment with 5 μM for 30 min and then Aβ1–42	Vehicle treatment	30-min	One-way ANOVA and Student’s t test, p-value	↓Intracellular Ca2 + levels ↓Mer Tyrosine Kinase decrease ↓NF-κB signaling ↓IL-1β and TNF-α	↓Alzheimer’s disease	Jhang et al. (2018)
Treatment of human microglia-like THP-1 cells with Aβ1–42	Pre-treatment with 5 μM for 30 min and then Aβ1–42	Pretreated with sulforaphane herbimycin A (1 μM), or Nrf2 activator (10 μM)	30-min	One-way ANOVA and Student’s t test, p-value	↓STAT-1 activation ↓IL-1β and miRNA-146a ↓NLRP3 inflammasome ↑HO-1 gene and Nrf2 levels	↓Alzheimer’s disease	An et al. (2016)
Aβ_42_ treatment in mice’s cortical neurons	Pre-treatment with 0.01, 0.03 or 0.1 μM for 30 min and then Aβ_42_	Six-month-old PS1V97L Tg105 mice (n = 6) were treated with corn oil	5 mg/kg 106 SFN in corn oil intraperitoneally 107 every day for 4 months until 10 months of age	Student’s t-test or the Mann–Whitney Utest, p-value < 0.05	↓Tau hyperphosphorylation ↓Dendritic integrity ↓Cell death	↓Alzheimer’s disease	Hou et al. (2018)

*p.o.* per os (by mouth), *i.p.* intraperitoneally

Dementia, characterized by cognitive decline as its primary symptom, presents significant challenges in the fields of public health, clinical practice, and policy. AD is the predominant form of dementia, accounting for around 60–70% of cases (Organization 2021). Despite extensive research conducted on risk and protective factors for dementia in high-income nations, projections indicate a significant shift by 2050. It is expected that a substantial majority, approximately three-quarters, of the projected 152 million individuals affected by dementia will reside in low- and middle-income countries (World Health Organization 2021). Consequently, even though treatments for AD exist, such as acetylcholinesterase inhibitors, N-methyl-D-aspartate (NMDA) receptor antagonists, and monoclonal antibodies like aducanumab and leqembi, these drugs cannot cure AD and cause side effects (e.g., brain swelling, microbleeds, fatigue, nausea, etc.), and a significant proportion of individuals in developing nations lack access to these interventions (Mintun et al. 2021; Salloway et al. 2022). The identification of novel therapeutics for AD is crucial to enhancing overall quality of life and facilitating the attainment of sustainable development goals (The Lancet Neurology 2023).

The application of genomics and proteomics research holds significant promise in unraveling the intricate interplay between genes, proteins, sulforaphane, and AD. These inquiries provide valuable pathways for comprehending specific molecular processes and identifying genetic and proteomic markers. Additionally, this methodology presents a comprehensive framework for deciphering the mechanisms through which sulforaphane bestows protection against AD. So, the primary objective of this study is to identify the molecular mechanisms potentially involved in the favorable effects of sulforaphane on AD.

## Materials and methods

### Link between sulforaphane and Alzheimer’s disease by the literature review approach

**Fig. 1:**
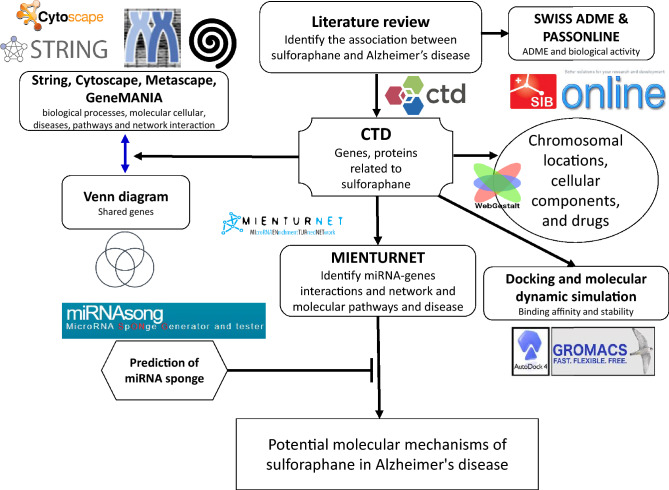
Detailed workflow for identifying and analyzing sulforaphane-related targets in Alzheimer’s disease

In order to investigate the potential relationship between sulforaphane and its involvement in the development of AD, a comprehensive review of the existing literature was conducted using multiple databases (Nguyen 2023). This study employed four distinct databases, namely ScienceDirect, PubMed, Google Scholar, and SpringerLink. The selection of these sources was based on the availability and accessibility of biomedical and life sciences literature pertaining to targeted metabolites and cognitive impairment, without any constraints. The subsequent search terms were utilized: “sulforaphane”, “DL-Sulforaphane”, “4478-93-7″, “1-Isothiocyanato-4-(methylsulfinyl)butane”, “D,L-Sulforaphane”, “molecular mechanism”, “genes”, “proteins”, “miRNAs”, “transcription factors”, “in vivo”, “in vitro”, memory deficits”, “memory loss”, “Alzheimer’s disease”, “cognitive dysfunction”, “cognitive deficits”, “dementia”, and “cognitive impairment”. The data collection spanned from January 1992 to October 8, 2023. A thorough evaluation of the literature was undertaken, spanning a diverse range of studies, including original research publications and review articles. These studies specifically investigated various aspects involving human beings, animals, and cellular models. The research demonstrating a correlation between sulforaphane and AD was incorporated. The workflow is outlined in detail in Fig. 1.

### Link between sulforaphane and Alzheimer’s disease by the in-silico approach

This study employed the following phrases to perform a search for correlations between sulforaphane and AD: “dementia,” “Alzheimer's disease,” “cognitive impairment,” “cognitive dysfunction,” “cognitive deficits,” “memory deficits,” and “memory loss.” The search was conducted utilizing the Comparative Toxicogenomics Database (CTD) (NC_State_University 2023). The examination described in this paper was conducted using the dataset acquired on October 8, 2023. Following this, through an examination of the “Direct Evidence” section, we have discerned the precise targets and sulforaphane compounds that are linked to the development of AD (VIB/UGent 2022). We initially investigated the gene–gene interaction network using GeneMANIA (http://genemania.org/plug-in/), a free plug-in for Cytoscape. The GeneMANIA Cytoscape plug-in provides valuable insights into various types of gene–gene interactions. These include physical interactions, where two gene products are linked based on protein–protein interaction studies, and co-expression, where genes show similar expression levels across conditions. Additionally, co-localization identifies genes or proteins that are expressed in the same tissue or found in the same location. Genetic interactions reflect functional associations, where the effect of perturbing one gene is influenced by changes to another. The tool also highlights pathway interactions, linking genes involved in the same biological pathways, and identifies shared protein domains, indicating common structural features in gene products. Together, these interaction types help predict and understand functional relationships among genes. In this analysis, Homo sapiens was selected as the target organism to explore interactions between genes. GeneMANIA helped identify related genes based on their co-expression, co-localization, genetic interactions, and other functional associations (University_of_Toronto 2022).

**Fig. 2:**
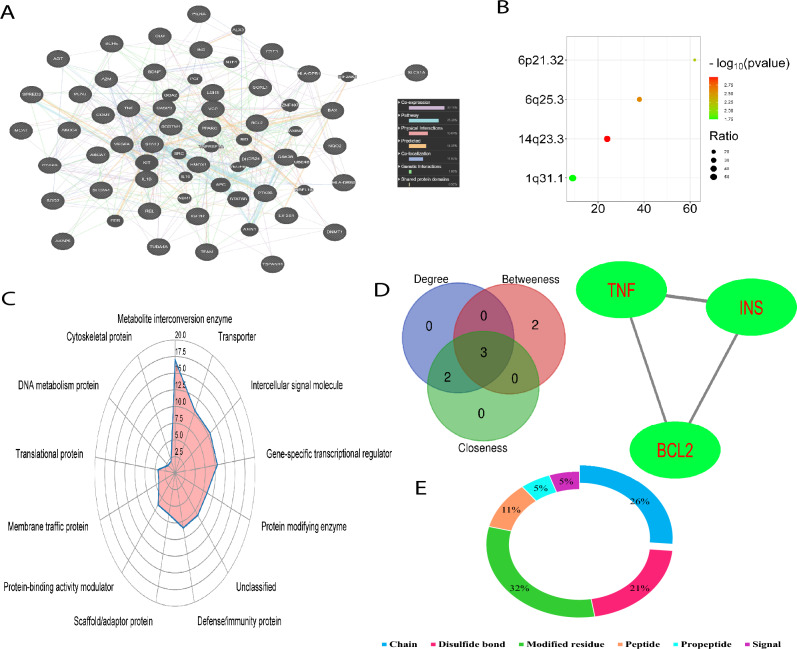
Network interaction and post-translational modifications of targets involved in AD pathogenesis regulated by sulforaphane. **A** Network interaction of the 45 sulforaphane-regulated targets, primarily characterized by co-expression (30.2%) and pathway interactions (25.4%), visualized using GeneMANIA (https://genemania.org). **B** Chromosomal locations of these targets, with 14q23.3 and 1q31.1 exhibiting the highest distribution ratios. **C** Functional classification of the targets, highlighting metabolite interconversion enzymes (17%), transporters (10%), intercellular signaling molecules (10%), and gene-specific transcriptional regulators (10%) based on Panther classification (http://www.pantherdb.org/). **D** Identification of hub targets (TNF, INS, and BCL2) through network topology analysis, utilizing degree, betweenness, and closeness scores. **E** Post-translational modifications (PTMs) of hub targets visualized using Uniprot (https://www.uniprot.org), with key modifications including modified residues (32%), chains (26%), and disulfide bonds (21%)

We utilized the CytoHubba plug-in to identify hub targets by focusing on three key topological parameters: degree, closeness, and betweenness. To begin, we constructed a protein–protein interaction (PPI) network for the target genes using the STRING V11 database, applying a composite score cut-off of 0.4 or higher (Chin et al. 2014). Cytoscape software was then employed to visualize the PPI network, based on data obtained from STRING. Through the CytoHubba plug-in in Cytoscape (http://apps.cytoscape.org/apps/CytoHubba), we conducted three topological analyses to detect hub genes, identifying the top 10 genes with the most significant interactions (Fig. 2).

Additionally, we have uncovered the underlying molecular processes through which sulforaphane effectively combats AD. The Metascape and Cytoscape software versions 3.9.1, along with the CytoscapeClueGO plug-in version 2.5.8, were utilized to examine biological processes, cellular function, pathways, transcription factors, and illnesses. The list of enrichments was extracted using the CytoscapeClueGO, Reactome, KEGG, and WikiPathways databases. The analyses conducted encompassed the investigation of illnesses, molecular pathways, biological processes, and protein–protein interaction enrichment (PPIE) analysis (Chen et al. 2009; Zhou et al. 2019). The species examined in this study was classified as Homo sapiens. Different databases, such as BioGrid (version 4.4.215), OmniPath, STRING (version 11.5), and InWeb IM, were used to study protein–protein interaction (PPI) networks. The present study examined the PPIE by utilizing physical interactions sourced from the STRING database. Specifically, the investigation focused on interactions that possessed a physical score over 0.4, as indicated by a previous publication (Zhou et al. 2019b). The network consists of proteins that participate in direct interactions with at least one additional component mentioned. The network is characterized by a protein count that spans from 3 to 500. In order to identify components within the network that exhibit a high level of interconnectedness, the Molecular Complex Detection (MCODE) technique is utilized (Zhou et al. 2019b). Further investigation was carried out on the pharmaceutical substances linked to the causation of AD and sulforaphane, utilizing WebGestalt, a web-based analytical tool (Liao et al. 2019a). The selected methodologies for the analyses encompass over-representative analysis, with a specific emphasis on pharmaceuticals, as well as the utilization of DrugBank and Illuminate Humanities 12 v4 databases (Liao et al. 2019b). The determination of the enrichment ratio in this investigation entailed dividing the observed count of pharmaceuticals discovered from the gene list by the anticipated count of drugs, as ascertained by the utilization of WebGestalt technologies. The proteoforms that arise as a consequence of post-translational modifications (PTMs) were ultimately discerned through the utilization of UniProt (The_UniProt_Consortium 2017). The protein class was categorized using the Panther classification approach through the assessment of targets (Thomas et al. 2003).

### miRNAs and miRNA sponges related to sulforaphane and Alzheimer’s disease

In this study, we employed the “MicroRNA ENrichment TURned NETwork” (MIENTURNET) tool to construct and analyze network diagrams that depict the associations between microRNA molecules and their respective targets (Licursi 2019). We performed functional enrichment analysis by utilizing the WikiPathway and illness ontology databases. A significance threshold of 0.05 was established in order to identify the functional annotations that demonstrated statistically significant enrichment within the entire gene group in the input set. The P-values underwent adjustment using the Benjamini–Hochberg method (Licursi et al. 2019). The miRNAsong online tool was employed to construct and evaluate the miRNA sponge templates associated with the target miRNAs activated by sulforaphane (Barta et al. 2016a). The sponge sequence exhibited two distinct regions that facilitated the binding of miRNAs: a bulge located at positions 26 and 27, and a spacer sequence denoted as AGAGG, which separated unique miRNA-binding sites. The results were obtained by employing a threshold of −20 kcal/mol and a canonical 7-mer seed (2–8) configuration. The secondary duplex pattern with minimum free energy was obtained using the RNAstructure web server. The three-dimensional structures were generated using the RNAComposer software and the BIOVIA Discovery Studio Visualizer program (Nguyen and Kim 2023b).

### Molecular docking

The present study investigates the interaction between sulforaphane and the hub targets. The RCSB protein database was used to get the crystal structures of hub genes, which were then used for molecular docking studies. In the current investigation, hub genes that demonstrated elevated amounts of x-ray crystallization were selected. The hub genes were created with MOE software (version 2014) prior to conducting the analysis. Water molecules and non-protein components were first excluded from the hub genes. The hub genes possess polar hydrogen atoms and Kollman charges as a means to minimize energy. The optimization of sulforaphane was conducted using the Avogadro program, utilizing the MMFF-94 force field and the steepest descent method for a total of 5000 iterations (Hanwell et al. 2012). In order to enhance the docking procedure between the hub genes and sulforaphane, the introduction of hydrogen atoms was implemented, followed by subsequent refinement of the ligand structures. The docking analysis was conducted with the AutoDock Vina software (version MGLTools 1.5.6) and the Discovery Studio 2016 program. In this study, several key terms related to molecular docking are defined. Grid size refers to the dimensions of the three-dimensional space surrounding the target protein that is evaluated during the docking simulation, ensuring that potential ligand interactions with key residues are comprehensively assessed. Exhaustiveness indicates the thoroughness of the docking search; a higher exhaustiveness value suggests a more extensive exploration of possible ligand positions, which can lead to improved predictions of binding affinity. Lastly, binding energy is the calculated energy change associated with the formation of the ligand–protein complex, where lower values typically signify stronger and more favorable interactions between the ligand and the target protein (Eberhardt et al. 2021). A value of eight was applied to the exhaustiveness parameter, whereas a value of four was assigned to the energy range parameter. These values were chosen based on their default settings in AutoDock Vina, which typically provides a good balance between accuracy and computational efficiency (Che et al. 2023; Feinstein and Brylinski 2015). The AUTOGRID technique is utilized to generate a three-dimensional grid in order to compute the cumulative binding energy between the analyzed metabolites and the central genes. The grid is generated in the form of a rectangular grid. The grid is configured with dimensions of 40 units along the x-axis, 40 units along the y-axis, and 40 units along the z-axis. Each point on the grid is separated by a distance of 0.375 Å (Nguyen and Kim 2022c).

### Molecular dynamic simulation

Molecular dynamic (MD) simulations were conducted using GROMACS 5.1.4 software and the charmm27 force field to assess the stability of the interaction between TNF and sulforaphane in terms of their binding affinity. The topology for the ligand under investigation was generated using the PRODRG2 online program (Abraham et al. 2015). Additionally, the study system’s boundaries were defined using a cubic simulation box, followed by the use of TIP3P water molecules to address the problem. Two chloride ions were introduced to achieve neutralization of the system under investigation. Subsequently, the occurrence of steric interactions was mitigated through the optimization of the simulated system via steepest descent minimization. To achieve system stability under optimal conditions, two equilibration processes were conducted. The first involved NVT equilibration (constant number of particles, volume, and temperature) utilizing a V-rescale thermostat at a temperature of 300 K for a duration of 1 ns. The second process involved NPT equilibration (constant number of particles, pressure, and temperature) using a Parrinello-Rahman barostat at a pressure of 1 atm, also for a duration of 1 ns (Bussi et al. 2007; Parrinello and Rahman 1981). Following the process of re-equilibration, the system underwent a molecular dynamics simulation for a duration of 100 ns. A range of statistics were obtained from the simulation results, including the root mean square deviation (RMSD), the number of hydrogen bonds, the radius of gyration (Rg), the solvent accessible surface area (SASA), and the root mean square fluctuation (RMSF). In our study, we utilized a RMSD cutoff of 2 Å to evaluate the stability of the ligand–protein complex during MD simulations, as this threshold is commonly accepted for distinguishing stable conformations (Fusani et al. 2020). The RMSD values remained below this cutoff, confirming the stability of interactions between sulforaphane and the target proteins. We employed the CHARMM27 force field, recognized for its extensive validation in protein–ligand interaction studies, as it provided accurate predictions for our system. Although we considered alternative force fields like AMBER, CHARMM27 demonstrated superior performance in similar contexts (Hornak et al. 2006). A simulation duration of 50 ns (ns) was chosen to adequately capture key interactions and potential conformational changes, as shorter simulations (10 ns or 20 ns) did not yield sufficient data regarding long-term stability (Pérez et al. 2007). This extended duration allowed for proper system equilibration and observation of relevant molecular motions.

### Analysis of drug likeness and absorption, distribution, metabolism, elimination, and toxicity (ADMET)

This study employed drug-likeness assessment to evaluate the suitability of sulforaphane for medicinal applications based on adherence to three key criteria: Veber’s, Igan’s, and Lipinski’s principles (Jayavel et al. 2024). The evaluation of medications targeting the central nervous system (CNS) involved the consideration of various factors, including the blood–brain barrier (BBB), the substrate for P-glycoprotein, the topological polar surface area (TPSA), and the number of rotatable bonds (n-ROTB). The drug-like qualities were assessed using the SwissADME webtool (Bioinformatics 2022). The evaluation of the features of the ADMET compounds being investigated was then conducted utilizing the AdmetSAR 1.0 web-based application (Cheng 2012). The assessment of absorption and distribution involved evaluating various factors, including human intestinal absorption (HIA), Caco-2 permeability, water solubility (LogS), and subcellular localization (Amaladoss et al. 2024). The investigation focused on the metabolic activity of the commonly occurring cytochrome P450 isoforms, including CYP2C19, CYP2D6, CYP3A4, and CYP1A1, among others (Jayavel et al. 2024). The evaluation of total clearance was conducted to determine its association with the process of secretion. The toxicological criteria assessed in this study encompassed various aspects, such as the toxicity of Salmonella/microsomes (AMES), acute oral toxicity, biodegradation, carcinogenicity, toxicity towards fish (pLC50 (negative logarithm of the lethal concentration for 50% of the population), mg/L), rats (LD50 (lethal dose for 50% of the population), mol/kg), and tetrahymena pyriformis (pIGC50 (negative logarithm (base 10) of the concentration of a drug that inhibits a biological process by 50% in an in vitro assay), ug/L).

### Analysis of biological activities

The biological effects of sulforaphane on AD were further examined by the utilization of the PASS online platform (PharmaExpert, Version 2.0) (Lagunin et al. 2000; Way2Drug 2011). After submitting sulforaphane to the PASS online platform, an assessment was conducted to determine its potential therapeutic effects, including several outcomes such as its anti-dementia capabilities, antioxidant properties, and others. The evaluation was carried out utilizing the probable activity (Pa) metric, which measures the probability of target metabolites demonstrating activity. The Pa values are expressed as a percentage (%) denoting probability and encompass a range from 0.0001 to 1.000 (Nguyen and Kim 2023a).

## Results

### The effects of sulforaphane on Alzheimer’s disease according to a literature review

Extensive research has been conducted on the potential neuroprotective properties of sulforaphane, particularly in relation to AD. Table 1 provides a summary of in vivo and in vitro models that investigate the effects of sulforaphane on memory impairment and pre-clinical biomarkers associated with AD, such as neuroinflammation, oxidative stress, tau hyperphosphorylation, amyloid-β, and neurodegeneration.

### Identification of targets underlying Alzheimer’s disease associated with sulforaphane

Using data from the CTD database, we identified 45 targets regulated by sulforaphane that are involved in the pathogenesis of AD (Table S1). GeneMANIA network analysis revealed that co-expression (30.2%) and pathway (25.4%) interactions were the predominant relationships among these targets (Fig. 1A). These targets were predominantly located on chromosomes 14q23.3 and 1q31.1, which exhibited the highest ratio of target distribution (Fig. 1B). The functions of these targets were categorized as metabolite interconversion enzymes (17%), transporters (10%), intercellular signal molecules (10%), and gene-specific transcriptional regulators (10%), indicating their diverse roles in biological processes related to AD (Fig. 1C). Through network topology analysis, utilizing parameters like degree, betweenness, and closeness scores, TNF, INS, and BCL2 emerged as the most critical hub targets in the sulforaphane-AD interaction network, further highlighting their pivotal roles in the disease’s molecular mechanisms (Fig. 1D and Figure S1). These targets are well-documented for their contributions to key AD pathways, including neuroinflammation (TNF), insulin signaling (INS), and apoptosis regulation (BCL2). Moreover, an analysis of the PTMs of these three hub targets (Fig. 1E) showed that modified residues (32%), chains (26%), and disulfide bonds (21%) were the most common PTMs observed in the interaction network. These modifications are crucial for protein function and stability, which can influence the progression of AD. Specifically, the PTMs of TNF, INS, and BCL2 may affect processes like protein activation, stability, and interaction capabilities, providing deeper insights into their role in the molecular underpinnings of AD (Figure S2 and Table S2).

Our subsequent analysis delved into the intricate interactions between sulforaphane and the shared targets (Table 2). This process involved a meticulous, manual examination of the “gene interactions” entries in the CTD database, where we gathered insights into how these interactions influence protein activity, mRNA expression, protein expression, and protein binding. We focused specifically on binary interactions—those representing a direct link between sulforaphane and one gene—while disregarding more complex interactions involving multiple molecules or genes, as well as interactions deemed to have "no effect." By concentrating on binary interactions, we could isolate the direct effects sulforaphane exerts on these genes, enhancing our understanding of its mechanism of action.

**Table 2:** The interactions between sulforaphane and targets related to Alzheimer’s disease (CTD Database, http://CTD.mdibl.org)

Parameters	Sulforaphane		
Interactions	Protein activity	Protein expression	mRNA expression
TNF	**–**	↓	↓
INS	–	↑	**–**
BCL2	**–**	↑	↑

↑ increase, ↓ decrease, *–* no expression

**Fig. 3:**
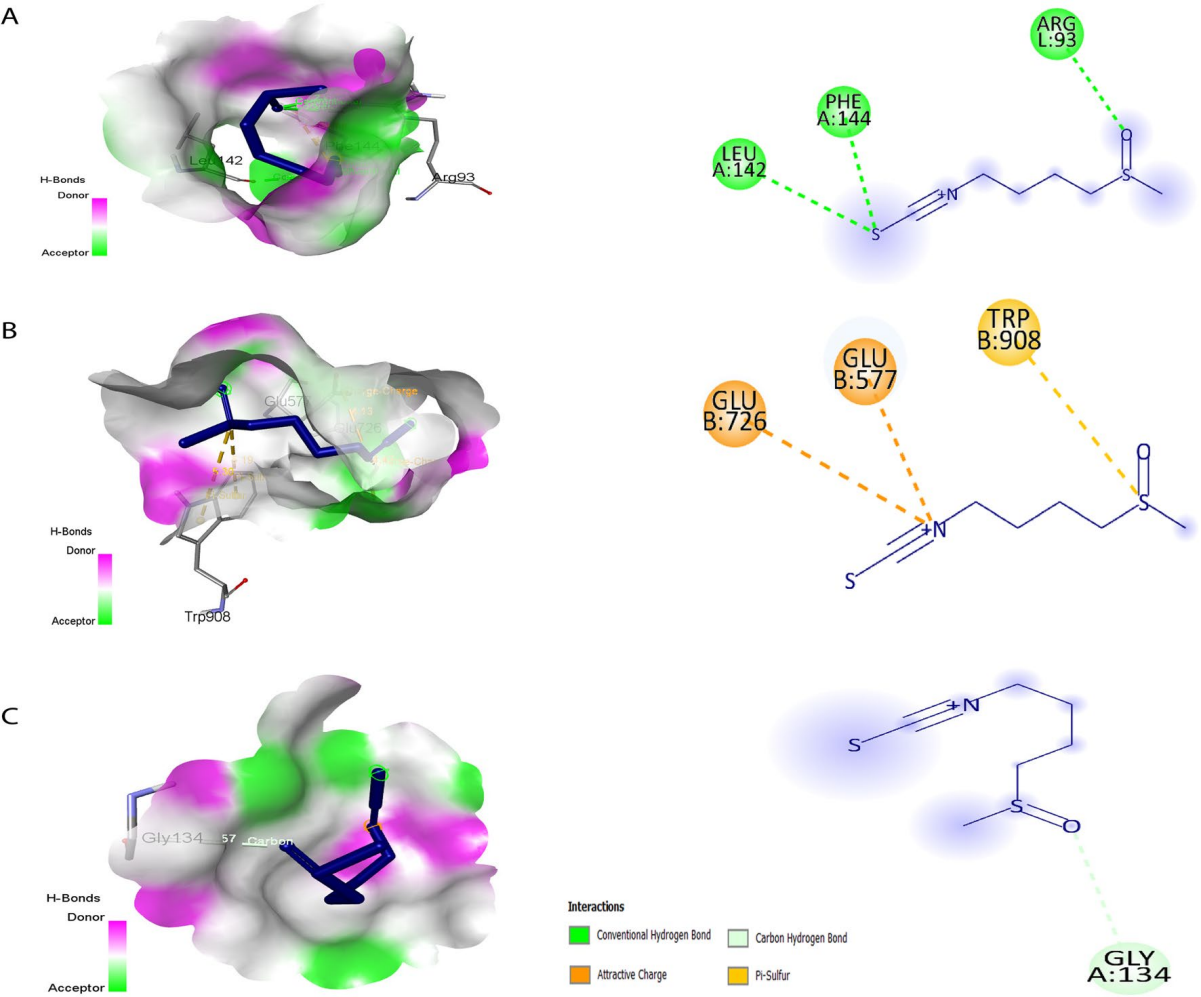
Direct interactions between sulforaphane and key targets involved in AD. **A** Sulforaphane’s interaction with TNF, illustrating its potential role in neuroinflammation. **B** Interaction with INS, suggesting implications for insulin signaling in cognitive function. **C** Interaction with BCL2, highlighting its relevance in apoptosis regulation. Each panel presents structural details to elucidate the binding mechanisms

The binding affinities of sulforaphane with key targets provide significant insights into its potential therapeutic mechanisms in AD. The docking scores indicate the strength of the interactions, with TNF exhibiting the most favorable score of −4.6 kcal/mol, suggesting a strong binding affinity (Table 3 and Fig. 3A−C). This robust interaction is facilitated by three critical hydrogen bonds with the amino acids Leu143, Phe144, and Arg93. The significance of TNF in neuroinflammation cannot be overstated, as elevated levels of TNF are associated with inflammatory processes that exacerbate neuronal damage and contribute to the progression of AD (Olmos and Lladó 2014). Therefore, the strong binding of sulforaphane to TNF may allow it to effectively modulate inflammatory pathways, potentially reducing neuroinflammatory responses and offering neuroprotective benefits. In comparison, the docking score for INS is slightly lower at −4.5 kcal/mol, which indicates a significant, but less robust interaction. INS is involved in insulin signaling pathways that are increasingly recognized for their role in cognitive functions and the pathophysiology of AD (Stanley et al. 2016). The interaction between sulforaphane and INS suggests a possible mechanism by which sulforaphane might enhance insulin sensitivity or promote neuronal survival through its effects on insulin signaling, warranting further exploration of its role in cognitive health.

**Table 3:** Interactions between sulforaphane and hub targets

Indicators	Ligand (ID PubChem)	ID RCSB	Center grid box	Docking score (kcal/mol)	Amino acids	Interactions
TNF	C_6_H_11_NOS_2_ (5350)	3WD5	x = −20.110 y = 21.623 z = 22.196	−4.6	Leu142, Phe144, Arg93	Conventional hydrogen bonds
INS		6BF6	x = 166.893 y = 188.880 z = 200.824	−4.5	Glu726, Glu577, Trp908	Attractive charge and Pi-sulfur
BCL2		1MAZ	x = 1.9350 y = 22.225 z = 39.274	−3.4	Gly134	Carbon hydrogen bond

The interaction with BCL2, which shows the lowest docking score of −3.4 kcal/mol, is particularly intriguing due to BCL2’s well-established function in regulating apoptosis and cell survival. This interaction suggests that sulforaphane could play a role in preventing neuronal cell death associated with AD, potentially providing a therapeutic avenue for mitigating neurodegeneration. By influencing the apoptotic pathways, sulforaphane may contribute to neuronal resilience, thereby addressing one of the hallmark features of AD pathology (Chernyuk et al. 2023). Taken together, our results highlight the biological implications of these interactions with sulforaphane, linking them to critical processes in AD pathology. This comprehensive analysis provides a clearer understanding of how sulforaphane may exert its beneficial effects in AD, emphasizing the need for further research to validate these interactions and their therapeutic potential.

**Fig. 4:**
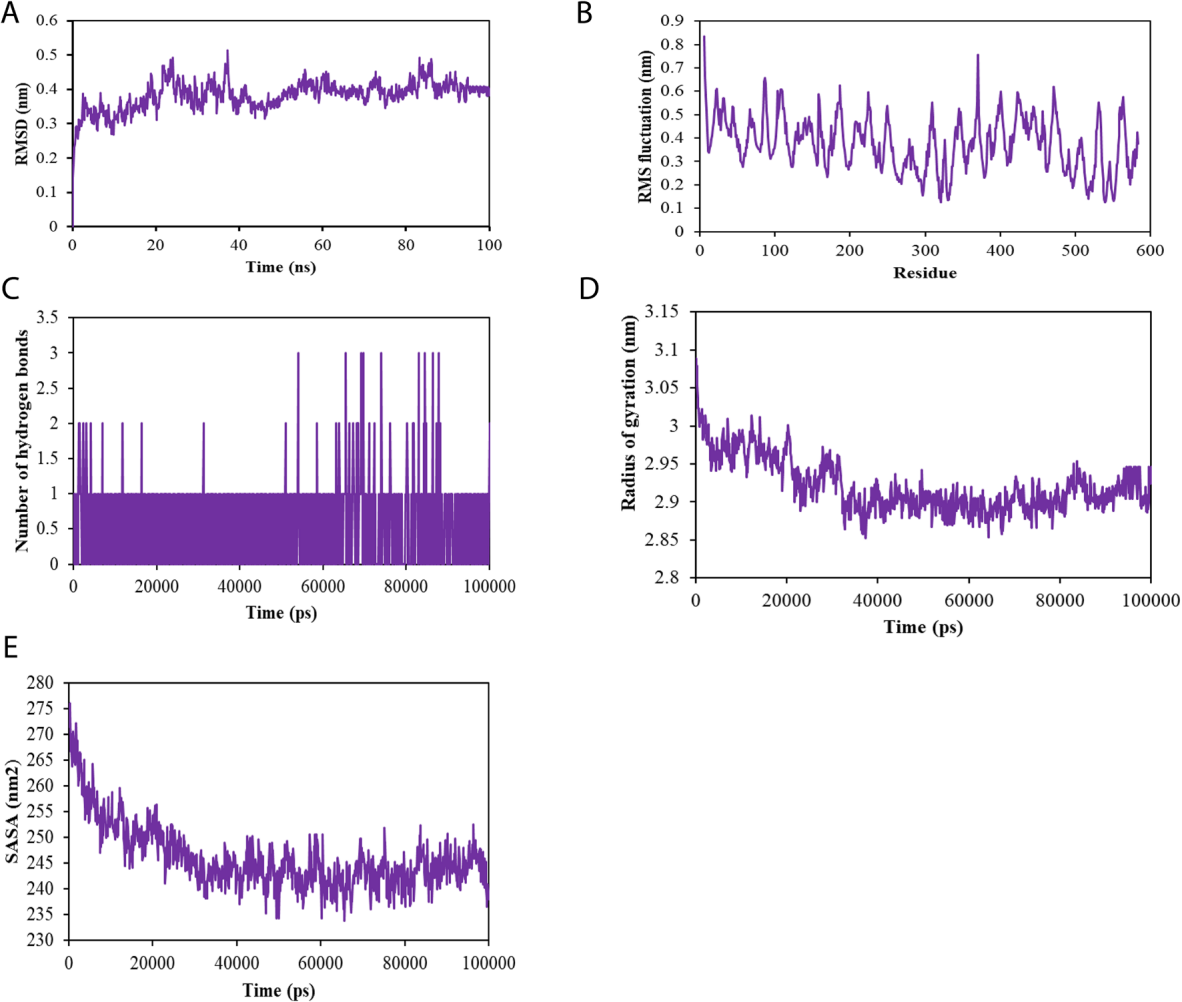
Molecular dynamics simulation of sulforaphane binding to tumor necrosis factor (TNF) associated with AD. **A** Root mean square deviation (RMSD) showing stability over time. **B** Root mean square fluctuation (RMSF) analysis of amino acid variations during the simulation. **C** Count of hydrogen bonds formed between sulforaphane and TNF, indicating binding strength. **D** Radius of gyration assessing the compactness of the TNF structure. **E** Solvent-accessible surface area (SASA) illustrating conformational stability of the sulforaphane-TNF complex throughout the simulation

The analysis presented in Fig. 4A–E provides critical insights into the stability of the sulforaphane-TNF complex during molecular dynamics simulations. The RMSD graph indicates a robust stability of the complex, with an average backbone RMSD of 0.375 nm and minimal fluctuations, suggesting that sulforaphane maintains a consistent interaction with TNF throughout the simulation period (Fig. 4A). This stability is essential for therapeutic applications, as it implies that sulforaphane can effectively bind to TNF without significant conformational changes that could disrupt its biological activity. Moreover, the RMSF analysis (Fig. 4B) reveals limited variation among the amino acid residues in the loop regions, with an average RMSF value of 0.4 nm. This minimal fluctuation indicates that the majority of TNF maintains its structural integrity during the interaction with sulforaphane, reinforcing the potential for sustained therapeutic effects. The consistent interaction between sulforaphane and TNF is further supported by the observation of stable hydrogen bonds throughout the 100-ns simulation (Fig. 4C). These persistent interactions enhance the likelihood that sulforaphane can effectively modulate TNF’s role in neuroinflammation, a key factor in AD pathology. The analysis of the radius of gyration (Fig. 4D) also confirms the protein’s steady folding, with limited fluctuations indicative of a stable structure. Additionally, the SASA assessment (Fig. 4E) predicts an average SASA value of 245 nm^2^ for the sulforaphane-TNF complex, suggesting that there were no significant conformational changes throughout the simulation. Together, these findings imply that sulforaphane’s stable binding to TNF, characterized by minimal fluctuations and consistent interactions, enhances its therapeutic potential in mitigating neuroinflammation associated with AD. This integration of figure results into the narrative emphasizes the significance of sulforaphane's interactions with TNF and their implications for therapeutic strategies targeting AD.

### Molecular mechanisms underlying Alzheimer’s disease are associated with sulforaphane

**Fig. 5:**
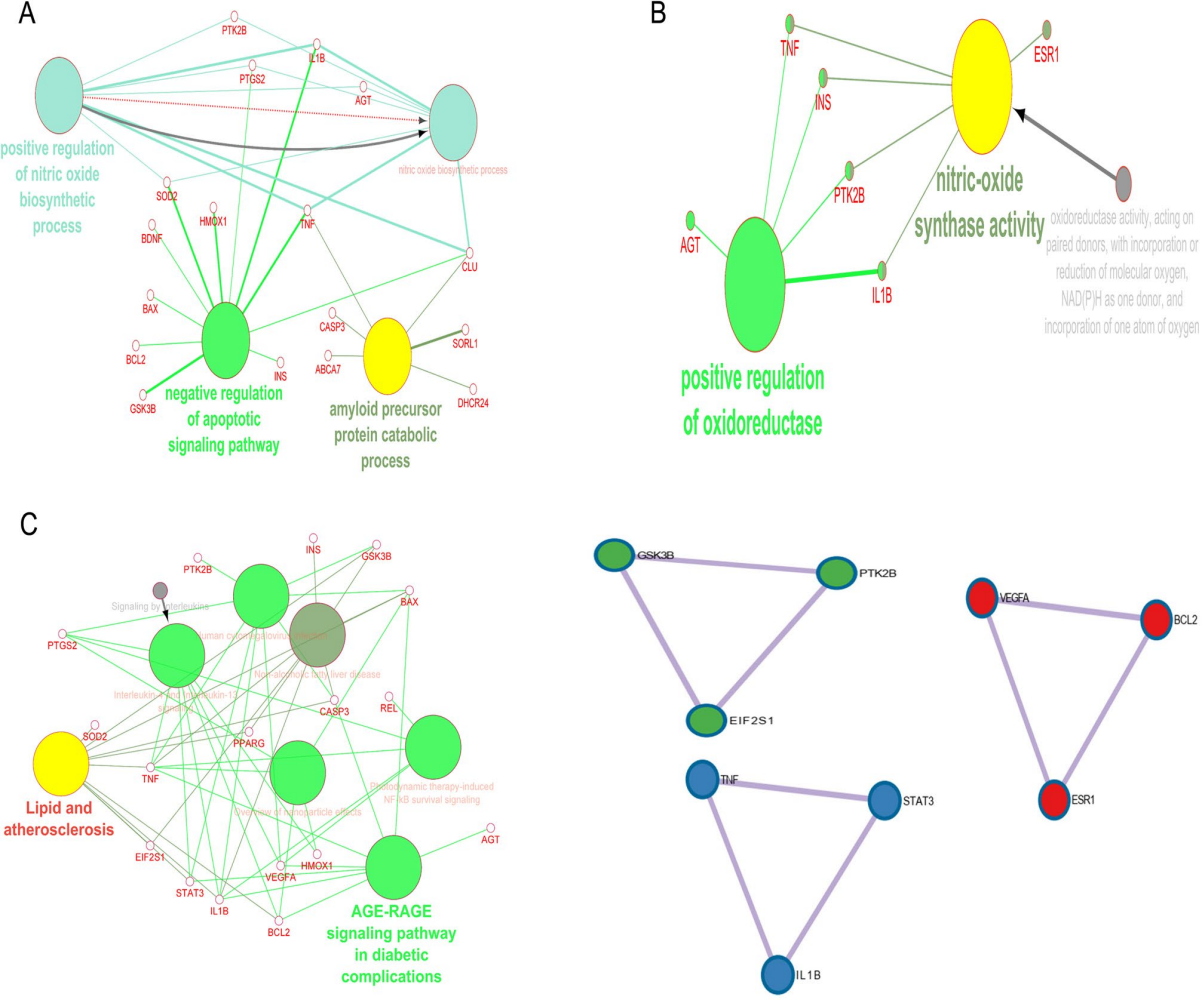
Biological processes, molecular functions, pathways, and protein–protein interactions (PPI) targeted by sulforaphane in AD. **A** Overview of key biological processes affected by sulforaphane, highlighting mechanisms related to nitric oxide synthesis and apoptosis regulation. **B** Molecular functions of the targets, indicating roles in enzymatic activity and signaling. **C** Key signaling pathways impacted by sulforaphane, relevant to AD pathogenesis. **D** PPI analysis revealing the interaction landscape among targets, performed using Cytoscape ClueGO and Metascape

To better understand how sulforaphane affects AD, we further analyzed the molecular mechanisms underlying the etiology of AD associated with sulforaphane. The targets related to AD and sulforaphane clustered around three biological processes (Fig. 5A), including “positive regulation of nitric oxide biosynthetic process”, “negative regulation of apoptotic signaling pathway”, and “amyloid precursor protein catabolic process”. For molecular function, “positive regulation of oxidoreductase” and “nitric oxide synthase activity” were denoted (Fig. 5B), while “lipid and atherosclerosis” and “AGE-RAGE signaling pathway in diabetic complications” were listed as key signaling pathways (Fig. 5C). PPIE analysis also highlighted “negative regulation of apoptotic signaling pathway” and “regulation of apoptotic signaling pathway” as the predominant signaling pathways implicated in the process of sulforaphane combating AD (Fig. 5D and Table S3). Furthermore, we also found other pathways regulated by sulforaphane that can ameliorate AD, such as “PID IL27 PATHWAY”, “PID IL23 PATHWAY”, “interleukin-10 signaling”, “brain-derived neurotrophic factor (BDNF) signaling pathway”, “protein autophosphorylation”, regulation of GTPase activity”, and “regulation of proteolysis”.

In order to gain a deeper comprehension of the involvement of sulforaphane in the therapeutic approach to AD, we conducted a process enrichment analysis on the specific target list (Figure S2). The targeted cells associated with AD and sulforaphane were identified as MANNO_MIDBRAIN_NEUROTYPES_HMGL (including serotonergic, neuroblast dopaminergic, dopaminergic neurons, GABAergic neurons, microglia, etc.). “Acute Confusional Senile Dementia”, “Alzheimer’s Disease, Focal Onset”, NFKB1, SP1, and RELA were found to be the most important diseases and transcription factors.

**Fig. 6:**
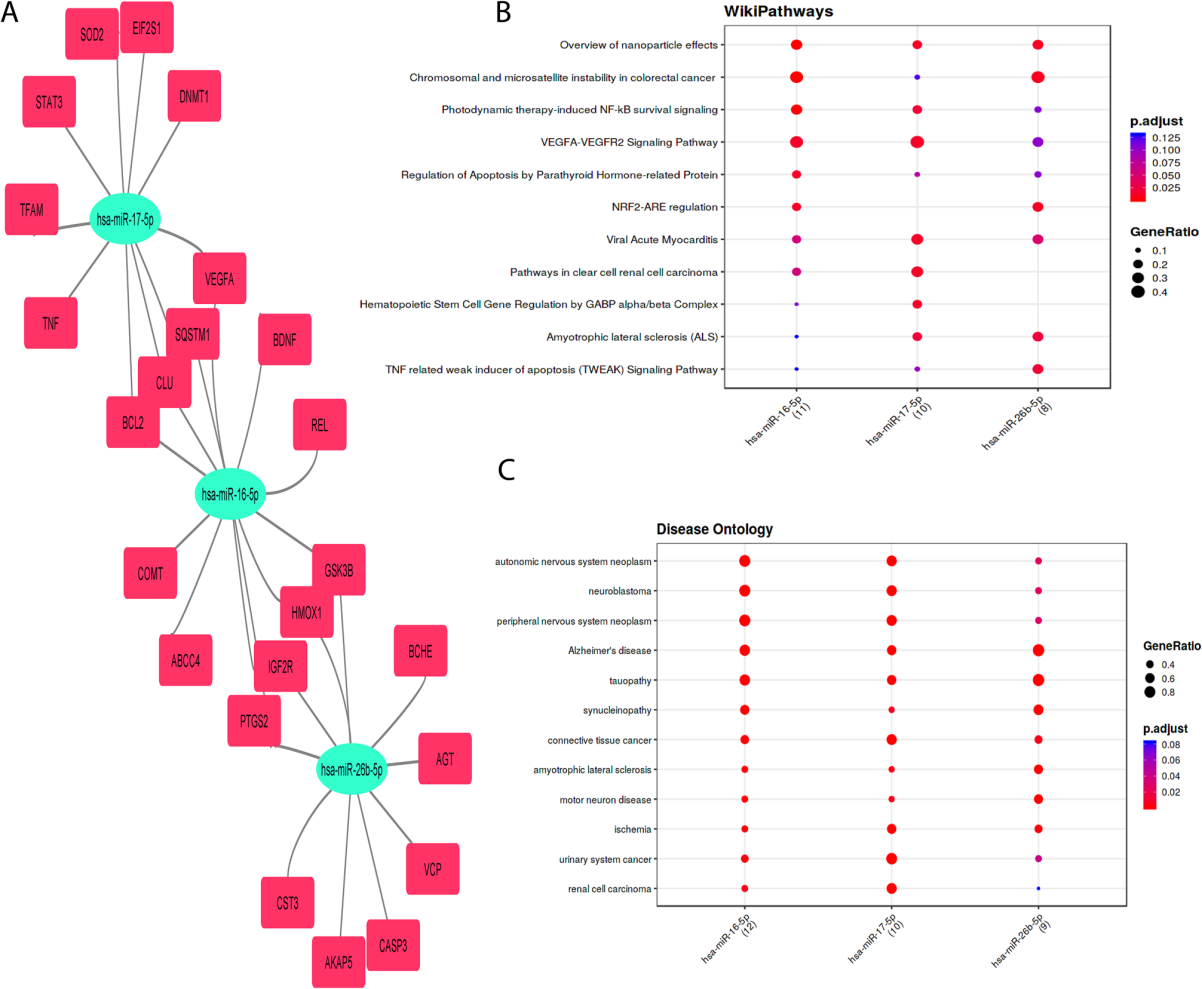
miRNA-target interactions relevant to AD and influenced by sulforaphane. **A** miRNA-target network depicting significant regulatory relationships among identified miRNAs and their target genes. **B** Signaling pathways associated with these interactions, highlighting their roles in AD pathology. **C** List of diseases linked to the identified miRNAs, demonstrating their broader relevance in neurodegeneration, analyzed using MIENTURNET

We further analyzed the interactions between target genes and miRNAs. In the network, node size reflects the degree of connectivity, with higher-degree miRNAs being more prominently associated with target genes (Khemka et al. 2024). In this study, key miRNAs were defined as those having strong connections with targets and the lowest adjusted p-values. Three key miRNAs—hsa-miR-17-5p, hsa-miR-16-5p, and hsa-miR-26b-5p—were identified as highly connected nodes within the target-miRNA regulatory network. The network comprised 27 nodes and 32 edges, with an average number of 2.37 neighbors per node. The network’s diameter was 6, and the radius was 3, indicating strong connectivity within the regulatory network. “Photodynamic therapy-induced NF-KB survival signaling”, “TNF related weak inducer of apoptosis signaling pathway”, “Alzheimer’s disease”, and “tauopathy” were annotated as important signaling pathways and diseases on how sulforaphane can protect against AD (Fig. 6A–C). The sponge structures of three important miRNAs and their interactions are shown in Figure S3. The sponges were utilized for in silico experimentation to assess off-target effects, with a range of free energy of duplex values ranging from −75.5 to −91.7 kcal/mol. In this study, we proceed to ascertain the primary pharmaceutical substances linked to 45 specific targets that exhibit potential for mitigating the progression of AD. Dexibuprofen exhibits a synergistic impact when combined with sulforaphane, making it a crucial drug in this context (Figure S4).

### Physicochemical properties, pharmacokinetics, and biological activities

**Fig. 7:**
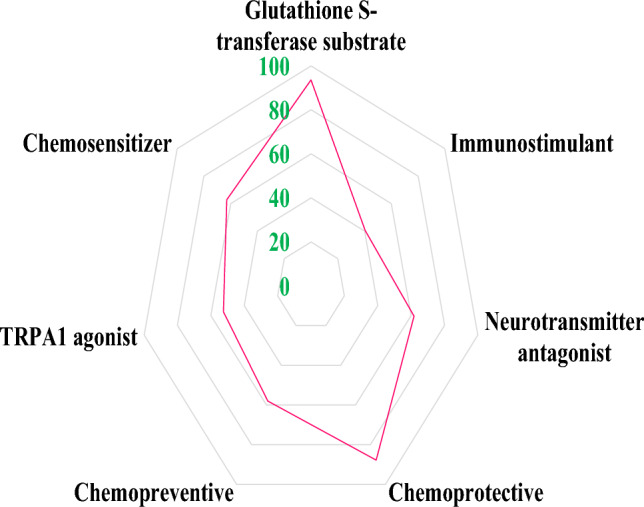
Biological activities of sulforaphane contributing to its protective effects against AD. This figure summarizes the pharmacological activities of sulforaphane, including its roles as an antioxidant, immunostimulant, and potential neuroprotective agent. Each biological activity is quantified, providing insights into how sulforaphane may ameliorate AD pathology, evaluated using PassOnline

To support the previous findings based on drug-like features of sulforaphane, we proceeded to evaluate the ADMET of sulforaphane. As seen in Table S4 and Figure S5, sulforaphane demonstrates a reasonable level of solubility in water and does not possess any toxicity or P-glycoprotein activity. Additionally, sulforaphane is very well absorbed in the human intestines and has the ability to pass through CaCO_2_ and cross the BBB. Sulforaphane did not exhibit inhibitory effects on CYP450 enzymes. The subcellular location of sulforaphane was shown to be predominantly in lysosomes. Collectively, these findings suggest that sulforaphane could potentially serve as an effective therapeutic agent for the treatment of AD. Similar to its physicochemical properties, sulforaphane exhibits several important biological activities that may ameliorate AD (Fig. 7). These include acting as a glutathione S-transferase substrate (93.6%), an immunostimulant (40.5%), a neurotransmitter antagonist (61.7%), a chemoprotective agent (87.7%), a chemopreventive agent (58%), a transient receptor potential ankyrin 1 (TRPA1) agonist (52.5%), and a chemosensitizer (62.9%).

## Discussion

Our findings observed that sulforaphane protects against AD by regulating 45 targets, especially TNF, ISN, and BCL2. These targets were found to be related to metabolite interconversion enzymes, modified residues, regulation of apoptotic signaling pathway, “Alzheimer’s Disease, Focal Onset”, NFKB1, SP1, and RELA, hsa-miR-17-5p, hsa-miR-16-5p, and hsa-miR-26b-5p. Furthermore, its physicochemical properties, pharmacokinetics, and biological activities support the efficacious effects of sulforaphane on AD, including druglikeness, BBB permeability, high intestinal absorption, glutathione S-transferase substrate (93.6%), immunostimulant (40.5%), neurotransmitter antagonist, and chemopreventive effects.

### Targets, chromosomes, protein classification, and post-translational modifications implicated in the pathogenesis of Alzheimer’s disease and targeted by sulforaphane

Our analysis pointed out 45 targets modulated by sulforaphane underlying the pathogenesis of AD. These targets were found focusing on the midbrain and “acute confusional senile dementia”, and “Alzheimer’s disease, focal onset”. In line with that, there is a strong link between changes in the structure of the dopaminergic midbrain and the development of noticeable behavioral symptoms in AD patients (D’Amelio et al. 2018). A case–control study with 50 AD patients and 50 healthy controls showed that AD patients had significantly more deformations in the upper posterior region of the brainstem, especially in the midbrain, compared to the control group (Lee et al. 2015).

The interactions among 45 targets were mostly characterized by co-expression and pathways. The findings of this study indicate that there is a potential linkage between the identified targets, provided that their expression levels exhibit similarity across different experimental settings. Additionally, it is suggested that the products of these targets may be related if they are involved in the same biochemical reaction within certain pathways (Nguyen and Kim 2022a). We also found 45 targets underlying AD pathogenesis associated with sulforaphane located in the chromosomes (14q23.3 and 1q31.1); these locations have been linked with the pathogenesis of AD (Cruts et al. 1996; Haltia et al. 1994; Tassano et al. 2014).

These targets related to sulforaphane and AD were predominantly in metabolite interconversion enzymes, and modified residues. Metabolite interconversion enzymes are a special group of enzymes that help change one metabolite (a small molecule involved in metabolic pathways) into another inside of cells (Cori 1972). The enzymes play a crucial role in the regulation and homeostasis of diverse metabolic pathways inside the human body. On the other hand, modified residues pertain to distinct segments of proteins that have undergone chemical alterations or modifications. These changes include a number of biochemical processes, like oxidation, phosphorylation, or acetylation, which add or take away certain chemical groups from the molecular structure of proteins (Uversky 2013). These alterations have the potential to impact the functionality and behavior of proteins. In the context of AD, it has been established that specific proteins undergo misfolding and aggregation, resulting in the development of detrimental plaques and tangles within the brain (Sultana et al. 2009; Sweeney et al. 2017). The presence of misfolded proteins has the potential to interfere with regular cellular functions and play a role in the development of AD (Sweeney et al. 2017). The connection between sulforaphane and these proteins potentially encompasses the prevention of aberrant modifications or facilitation of the degradation of detrimental metabolites, hence mitigating the pathogenic mechanisms linked to AD. Our analysis also found “protein autophosphorylation”, “regulation of GTPase activity”, and “regulation of proteolysis” were listed as the most important signaling pathways underlying AD pathology and targeted by sulforaphane. Thus, through its modulation of these particular proteins, sulforaphane has the potential to exert a regulatory effect on cellular processes, hence potentially attenuating the advancement of AD.

### Key targets implicated in the pathogenesis of Alzheimer’s disease and targeted by sulforaphane

In this analysis, TNF, INS, and BCL2 were annotated as the most important targets based on network topology analysis. The cytokine tumor necrosis factor (TNF) plays a crucial role in the processes of inflammatory responses by activating immune cells through its receptor, TNF receptor 1 (TNFR1). Furthermore, the activation of reactive oxygen species and reactive nitrogen species-generating enzymes might lead to the direct induction of oxidative stress by TNFR1. The interaction between TNF-induced oxidative stress and inflammation has been widely acknowledged as contributing factors to the development of neurodegeneration, particularly in the context of AD (Chang et al. 2017a; Fischer and Maier 2015). The administration of sulforaphane to rats with AD-like symptoms produced by Aβ resulted in a decrease in neuroinflammation, as evidenced by reductions in the levels of TNF-α and IL-1β (Hou et al. 2018; Wang et al. 2020). Sulforaphane protects against neuronal necroptosis induced by lipopolysaccharide by reducing the mRNA and protein levels of pro-inflammatory mediators (TNF-α, IL-1β, IL-6, and iNOS) in BV-2 microglia (Table 1) (Qin et al. 2018). The p75 neurotrophin receptor is a transmembrane protein found within the TNF receptor family. This receptor has been associated with the development of AD. In both in vivo and in vitro investigations, it has been observed that sulforaphane exhibits the ability to improve neurobehavioral impairments and decrease the accumulation of Aβ in mouse models of AD (Zhang et al. 2017). The underlying mechanism responsible for these effects appears to involve the up-regulation of the p75 neurotrophin receptor, which is mediated, at least partially, through the reduction of histone deacetylase 1 and 3 expression (Zhang et al. 2017). This study also observed that sulforaphane reduced the protein and mRNA expression of TNF.

Diabetes is a prevalent metabolic disorder that is distinguished by elevated blood glucose levels, hyperinsulinemia, and insulin resistance. Oxidative stress associated with diabetes is also a contributing factor to cognitive impairments and the development of AD (McCrimmon et al. 2012). Sulforaphane serves as a pharmacological agent that activates the nuclear factor erythroid 2-related factor 2 (Nrf2), hence stimulating intracellular defense mechanisms mediated by Nrf2. These mechanisms encompass antioxidant and anti-inflammatory responses, which are particularly effective in combating oxidative stress (Pu et al. 2018). In mice fed a high-fat diet, the administration of varying doses of sulforaphane (2 and 10 mg/kg) resulted in several physiological effects. These effects included an increase in serum insulin levels, an improvement in the HOMA-β index, a reduction in fasting blood glucose levels, as well as decreases in serum low-density lipoprotein triglyceride, total cholesterol, and fibroblast growth factor 21 levels, implying sulforaphane may alleviate non-alcoholic fatty liver disease and facilitate the repair of pancreatic tissue (Tian et al. 2021). The findings of the in vitro study indicate that sulforaphane has the ability to restore glucose homeostasis and enhance insulin sensitivity by inhibiting the production of ceramide through the modulation of SPTLC3 in insulin-resistant HepG2 cells (Teng et al. 2019). An in vivo study demonstrated that sulforaphane exhibited a notable and dose-dependent reduction in the impairments generated by streptozotocin-induced diabetes, including endothelial, biochemical, and behavioral parameters (Sharma et al. 2021). In line with that, this analysis also found that sulforaphane increased INS protein expression.

The etiology of AD involves the activation of Aβ1–42, which induces a rapid and prolonged decrease in the expression of B cell lymphoma-2 (BCL-2), an important protein that inhibits cell death (Paradis et al. 1996). Furthermore, the crucial involvement of the multidomain proapoptotic BCL2-Associated X (BAX) and BCL2-2 homologous antagonist/killer (BAK) in the initiation of apoptosis with sulforaphane has been well documented (Choi and Singh 2005). Pretreatment with sulforaphane at concentrations ranging from 1 to 5 μM has shown a protective effect on the cells, mitigating cytotoxicity and apoptosis. SFN exhibited a decrease in apoptosis through the upregulation of BCL-2 and the inhibition of c-Jun N-terminal kinase activation and the proapoptotic protein BAX and oxidative stress in SH-SY5Y cells that were caused by Aβ25–35 (15 μM) (Lee et al. 2013). Additionally, another study has demonstrated that sulforaphane has the ability to reestablish the binding of acetyl-histone H3 to the BCL-2 promoter, thereby preventing apoptosis in neural crest cells and mouse embryos after exposure to ethanol (Yuan et al. 2018). In this analysis, we also observed the expression of sulforaphane mRNA and protein. Taken together, these findings suggest that sulforaphane has efficacious effects on AD by regulating neuroinflammation, insulin resistance, and apoptosis.

Given the critical roles of TNF, INS, and BCL2 in AD pathology, it would be valuable to incorporate docking scores or simulation results that specifically examine the interactions of these targets within the context of AD. For instance, previous studies have demonstrated that TNF receptor interactions can lead to downstream signaling pathways associated with neuroinflammation and apoptosis (Jayaraman et al. 2021; Webster and Vucic 2020). By comparing our findings with established data on TNF interactions, we can gain a clearer understanding of the ligand-receptor dynamics and their implications for neurodegeneration. Furthermore, analyzing INS and its interactions through molecular docking could shed light on how insulin signaling pathways are disrupted in AD, especially considering the links between insulin resistance and cognitive decline (Cui et al. 2022). Similarly, evaluating BCL2 interactions could elucidate the mechanisms by which sulforaphane enhances cell survival and reduces apoptosis in the presence of Aβ (Schepici et al. 2020). Integrating these docking studies into our analysis not only strengthens the foundation of our findings but also aligns them with existing literature, highlighting the therapeutic potential of targeting these pathways. This approach will ultimately provide a more robust framework for understanding the multifaceted roles of these proteins in AD and the potential for sulforaphane as a treatment.

### Key signaling pathways implicated in the pathogenesis of Alzheimer’s disease and targeted by sulforaphane

Our study highlighted the therapeutic effects of sulforaphane on AD by regulating the apoptosis signaling pathway, the “brain-derived neurotrophic factor (BDNF) signaling pathway”, “lipid and atherosclerosis”, and the “AGE-RAGE signaling pathway in diabetic complications”. Coinciding with previous literature, the administration of sulforaphane at a dosage of 0.5 mg/kg prior to treatment had a mitigating effect on the death of hippocampal neurons caused by chronic intermittent hypoxia (reduced cleaved PARP and cleaved caspase 3, and elevated BCL-2) (Li et al. 2022). In diabetic models, sulforaphane (orally 25 mg/kg) reduced caspase-3 and myeloid cell leukemia 1 expression (an anti-apoptotic Bcl-2 family member), as well as elevated p-Akt, p-GSK3β, nerve growth factor, and BDNF levels in diabetic rats, indicating potential for preventing memory impairment and death of hippocampus neurons (Wang et al. 2016). Oral sulforaphane (5 to 15 mg/kg) treatment in ApoE-deficient (ApoE − / −) mice that were fed a high-fat diet resulted in the amelioration of dyslipidemia, the formation of atherosclerotic plaques, and the unstable phenotype, which is known to be a risk factor for AD (Liu et al. 2023).

The production of neurotoxic Aβ peptides from APP proteolysis is critical to AD development. In AD, Aβ and hyperphosphorylated/cleaved tau forms were found to build up in the brain (O’Brien and Wong 2011). As shown in Table 1, numerous in vivo and in vitro studies have demonstrated the efficacious effects of sulforaphane on AD by reducing the accumulation of Aβ and its toxicity. In a recent study, sulforaphane (1 µM) treatment has been shown to mitigate neuronal damage caused by microglia through the down-regulation of the ROS/autophagy/NLRP3 signaling pathway in microglia stimulated by fibrillar Aβ (Yang et al. 2023). This study also found that sulforaphane regulated the “amyloid precursor protein catabolic process” to combat AD.

The involvement of nitric oxide in neuroinflammation is believed to be attributed to its free radical characteristics, which have the potential to negatively impact cellular integrity and viability through the induction of mitochondrial damage and contribute to the development of AD (Togo et al. 2004). Our study found that sulforaphane can modulate the “positive regulation of nitric oxide biosynthetic process”, “positive regulation of oxidoreductase” and “nitric oxide synthase activity”, which are important processes for ameliorating AD. In line with that, in an in vivo study, sulforaphane was observed to reduce inducible nitric oxide synthase (iNOS), proinflammatory levels (IL-1β, IL-6), cyclooxygenase-2 (COX-2), and nuclear factor (NF)-κB p-p65 in mouse neuroblastoma N2a cells expressing APPswe (Zhao et al. 2018).

IL-10, IL-23, and IL-27 play important roles in the pathogenesis of AD (Guillot-Sestier et al. 2015; Nitsch et al. 2021; Nortey et al. 2022). In an in vivo study, the administration of sulforaphane at a dosage of 50 mg/kg demonstrated a beneficial effect on the progression of experimental autoimmune encephalomyelitis in mice. This effect is achieved through the antagonism of oxidative stress and the reduction of Th17-related inflammation while simultaneously increasing the levels of IL10 (Li et al. 2013). At the peripheral level, it has been observed that sulforaphane exhibits a protective effect against T-cell-mediated autoimmune illnesses. This protective mechanism is achieved by the suppression of IL-23 and IL-12 in dendritic cells, as well as the antagonism of Th17-related inflammation in mice (Hernández-Rabaza et al. 2016). In our analysis, “PID IL27 PATHWAY”, “PID IL23 PATHWAY”, and “interleukin-10 signaling” were denoted as important signaling pathways underlying the pathogenesis of AD and targeted by sulforaphane.

### Key miRNAs and transcription factors implicated in the pathogenesis of Alzheimer’s disease and targeted by sulforaphane

Sulforaphane, an isothiocyanate from cruciferous vegetables, influences multiple molecular targets, including miRNAs, transcription factors, and epigenetic regulators. Studies demonstrate that sulforaphane modulates key pathways like Nrf2, apoptosis, and inflammation by regulating miRNAs such as let-7a-5p, miR-200c, and miR-21, which are involved in cancer and oxidative stress (Dacosta and Bao 2017). Using bioinformatics tools (e.g., MIENTURNET, miRWalk, TargetScan), we predicted sulforaphane’s impact on miRNA-target interactions and conducted pathway enrichment analyses using WikiPathway and KEGG. These approaches allow for a systematic understanding of its effects on cellular processes. By combining experimental data with in silico modeling, we can predict novel miRNAs and targets, expanding the known scope of sulforaphane’s biological impact.

miRNAs and transcription factors are significant contributors to the pathogenesis of AD (Nguyen and Kim 2022b). A study of human temporal lobe brain tissue sections from AD patients and age-matched controls without dementia discovered that hsa-miR-17-5p was higher in AD microglia around Aβ deposits compared to non-disease controls (Estfanous et al. 2021). The upregulation of miR-16-5p in the brain tissue of 5xFAD mice is induced by amyloid-β deposition. This upregulation leads to the induction of neuronal cell death by the direct targeting and reduction of BCL-2 (Kim et al. 2020). In an in-silico analysis using the Gene Expression Omnibus (GEO) database, miR-26a-5p expression was found to be upregulated in individuals with AD in comparison to control subjects (Chang et al. 2017b). This study also found that hsa-miR-17-5p, hsa-miR-16-5p, and hsa-miR-26b-5p, “photodynamic therapy-induced NF-KB survival signaling”, “TNF related weak inducer of apoptosis signaling pathway”, “Alzheimer’s disease”, and “tauopathy” were listed as the predominant miRNAs, signaling pathways, and diseases involved in AD and sulforaphane. On the other hand, NFKB1, SP1, and RELA were also listed as the key transcription factors underlying AD pathogenesis and targeted by sulforaphane in this study. Coinciding with these findings, numerous studies have also reported the effects of sulforaphane on AD by regulating the NFKB signaling pathway (Table 1).

### miRNA sponges and drugs implicated in the pathogenesis of Alzheimer’s disease and targeted by sulforaphane

miRNA sponges have been extensively employed in studies investigating the loss-of-function of miRNAs (Barta et al. 2016b). miRNA sponges are RNA transcripts that possess many binding sites with high affinity, enabling them to effectively attach to specific miRNAs. This binding interaction hinders the miRNAs from forming associations with their target mRNAs, hence reducing their regulatory function (Ebert and Sharp 2010; Nguyen and Kim 2023b). In an in vitro study, it was observed that the suppression of high miR-17 in the microglia of 5xFAD mice leads to enhancements in autophagy, Aβ degradation, and the generation of neighboring BRCA1 gene 1 puncta, suggesting the potential use of treatment strategies aimed at enhancing autophagy function in individuals with AD (Estfanous et al. 2021). As mentioned above, there was a link between overexpression of hsa-miR-17-5p, hsa-miR-16-5p, and hsa-miR-26b-5p and AD pathogenesis (Chang et al. 2017b; Estfanous et al. 2021; Kim et al. 2020). Thus, we designed and tested miRNA sponge structures for these miRNAs using miRNAsong in silico, which had the highest interactions with targets linked to the pathogenesis of AD that were linked to sulforaphane. These findings will pave the way for subsequent investigations to consider including these findings when examining the correlation between sulforaphane, miRNAs, sponges, and AD.

On the other hand, our study revealed the presence of dexibuprofen (ibuprofen’ active enantiomer and a nonsteroidal anti-inflammatory drug), a substance that has the potential to have a synergistic effect when co-administered with sulforaphane. Numerous studies have also reported the effects of dexibuprofen on AD. For example, the administration of dexibuprofen at a dosage of 20 mg/kg/day for a duration of 3 months has been shown to improve both peripheral and central risk variables that are commonly linked with AD in metabolically challenged APPswe/PS1dE9 mice (Ettcheto et al. 2021). An additional in vitro investigation has also demonstrated that the administration of Dexibuprofen at a dosage of 50 mg/kg effectively mitigates microglial activation and the resulting impairments in spatial working memory caused by the prolonged infusion of lipopolysaccharide in male Wistar rats (Jin et al. 2008). Remarkably, an in vitro investigation examined the potential synergistic impact of combining ibuprofen and sulforaphane on the down-regulation of DNA binding activity of the p50 subunit of NF-κB and the subsequent reduction in cell viability in human pancreatic cancer Panc-1 and MIA PaCa-2 cells. The findings of this study suggest that this combination therapy holds promise as a potential approach for both the prevention and treatment of pancreatic cancer (Thakkar et al. 2015).

### Physicochemical properties, pharmacokinetics, and biological activities implicated in the pathogenesis of Alzheimer’s disease and targeted by sulforaphane

Sulforaphane satisfies the drug-like criteria established by Veber, Lipinski, and Egan. Sulforaphane exhibited non-toxic properties and demonstrated favorable oral absorption (Tahata et al. 2018). Sulforaphane is well distributed and transported through the lysosomal compartments. It has been observed that sulforaphane, a compound that is capable of permeating the BBB and is not P-glycoprotein, exhibits positive effects in the context of AD (Greco and Fiskum 2010; Luis-García et al. 2017; Marek-Trzonkowska et al. 2012). Furthermore, sulforaphane exhibited superior distribution indices. In order to assess the stability of sulforaphane as a pharmaceutical prior to excretion, an evaluation of its excretion properties is conducted. The prediction calculations indicate that the total clearance index of sulforaphane is 7.7, suggesting that it has the potential to persist in the body and carry out its biological activities (Kikuchi et al. 2021). The inhibition of CYP450 enzymes has the potential to modify the metabolism of medications and thereby counteract their intended effects. In this study, it was observed that sulforaphane did not exhibit inhibitory effects on CYP450 enzymes (Kikuchi et al. 2021). This finding suggests that sulforaphane’s medication metabolism is deemed acceptable since it can effectively reach the therapeutic target without undergoing oxidation and subsequent elimination. Sulforaphane has demonstrated significant biological activities that have the potential to improve AD, including acting as a substrate for glutathione S-transferase, stimulating the immune system, antagonizing neurotransmitters, providing protection against chemical damage, preventing the onset of cancer, activating transient receptor potential ankyrin 1 (TRPA1) receptors, and enhancing the sensitivity of cells to chemotherapy. These biological activities have been reported as important properties for AD management (Choi and Singh 2005; Hou et al. 2018; Li et al. 2022; Yang et al. 2023; Zhao et al. 2018). Taken together, these findings imply that sulforaphane may indeed be a potent compound for AD treatment.

### Limitations

The present study elucidates the molecular mechanisms underlying the advantageous effects of sulforaphane in AD. Nevertheless, the present study does possess several limitations. The quality of analysis derived from online sources is contingent upon the relevance of the available data, given the inherent limitations of such sources in terms of the quantity of information they provide. The evaluation did not include an assessment of the dose–response relationship, mode of exposure, and period of sulforaphane exposure, nor did it include the specific sensitivities of the subjects under investigation. These factors are crucial for understanding the pharmacodynamics and pharmacokinetics of sulforaphane, and their absence limits the translational applicability of our findings. The findings of this study were consistent with prior research, as they substantiated the potential impact of sulforaphane on AD. Additionally, while computational predictions can provide valuable insights, they often lack the experimental validation needed to confirm biological relevance. In silico methods, by their nature, can be susceptible to assumptions and simplifications that may not accurately reflect physiological conditions. Therefore, relying solely on these models without empirical data can lead to overconfidence in their predictions. Furthermore, potential biases in the available datasets, such as variations in experimental design, sample sizes, and methodologies, could affect the robustness of our conclusions. Future studies should aim to include a more diverse range of data and validate findings through experimental approaches. Despite these limitations, our findings are consistent with prior research and substantiate the potential impact of sulforaphane on AD. They may offer valuable insights into the molecular underpinnings that can inform future in vivo and in vitro research endeavors. Addressing these limitations in future studies will be essential for fully understanding the therapeutic potential of sulforaphane and for developing effective treatment strategies for AD.

To build upon our findings, future research should prioritize in vitro and in vivo validation to confirm the roles of the identified target genes, miRNAs, and TFs in the context of AD. Specifically, in vitro studies could involve the use of neuronal cell lines or primary neurons subjected to AD-like conditions, where the effects of sulforaphane on the expression levels of TNF, INS, and BCL2 can be directly measured. Such studies would allow for a clearer understanding of the dose–response relationships and cellular mechanisms by which sulforaphane exerts its neuroprotective effects. In vivo validation is also crucial; experiments using appropriate animal models of AD can help elucidate the therapeutic potential of sulforaphane in a more complex biological environment. For instance, assessing the impact of sulforaphane on neuroinflammation, oxidative stress markers, and cognitive function in these models would provide essential insights into its efficacy and mechanisms of action. Moreover, our findings suggest the involvement of specific signaling pathways, such as the regulation of apoptotic signaling and nitric oxide biosynthesis. Future studies should investigate these pathways to confirm their modulation by sulforaphane and to identify downstream effects on neuronal survival and function. Additionally, the interplay between sulforaphane and key miRNAs—such as hsa-miR-17-5p, hsa-miR-16-5p, and hsa-miR-26b-5p—should be explored to understand how these molecules contribute to the compound’s therapeutic effects. By addressing these areas, future experimental designs can be more effectively informed, leading to a comprehensive understanding of sulforaphane’s role in AD and paving the way for its potential application in therapeutic strategies.

## Conclusion

This study observed that sulforaphane may ameliorate AD by regulating 45 targets, especially TNF, INS, and BCL2, suggesting the importance of the effects of sulforaphane on neuroinflammation, insulin resistance, and apoptosis on AD pathogenesis. Co-expression and pathways were the key interactions. 45 targets were found to be related to chromosomes (14q23.3 and 1q31.1), the midbrain, metabolite interconversion enzymes, and modified residues. “Amyloid precursor protein catabolic process”, regulation of apoptotic signaling pathway”, and “positive regulation of nitric oxide biosynthetic process” were the main pathways, while NFKB1, SP1, and RELA, hsa-miR-17-5p, hsa-miR-16-5p, and hsa-miR-26b-5p were key transcription factors and miRNAs implicated in sulforaphane protective effects against AD. The potential therapeutic efficacy of miRNA sponges and dexibuprofen, in conjunction with the co-administration of sulforaphane, has been identified in the treatment of AD. On the other hand, the therapeutic benefits of sulforaphane in AD can be attributed to its specific physicochemical properties, pharmacokinetic features, and biological activities. These advantages are a result of its unique characteristics, including efficient absorption within the gastrointestinal tract, possessing drug-like properties, absence of inhibition of CYP450 enzymes, not being a substrate for P-glycoprotein, ability to traverse the blood–brain barrier, serving as a substrate for glutathione S-transferase, exhibiting immunostimulant effects, antagonistic actions on neurotransmitters, and possessing properties that offer protection against chemical-induced damage. These distinct attributes align with sulforaphane’s therapeutic potential in the context of AD. In conclusion, while our findings position sulforaphane as a promising candidate for AD management, future studies are essential to validate these results through clinical trials. A comprehensive understanding of its pharmacological effects and the interplay of the identified molecular targets will be crucial in developing effective therapeutic strategies for AD.

## Supplementary Information

Below is the link to the electronic supplementary material.

Supplementary file1 (DOCX 2948 KB)

## Data Availability

No datasets were generated or analysed during the current study.
