# Supplementary material for: Molecular mechanisms of sulforaphane in Alzheimer’s disease: insights from an in-silico study
Source: In Silico Pharmacol. 2024 Nov 1;12(2):96. doi: 10.1007/s40203-024-00267-4 (PMC11530583; doi:10.1007/s40203-024-00267-4)
Supplement: Supplementary file 1 — Supplementary file1 (DOCX 2948 KB) [file 40203_2024_267_MOESM1_ESM.docx]

**Supplementary data**

| **A** |
| --- |
| 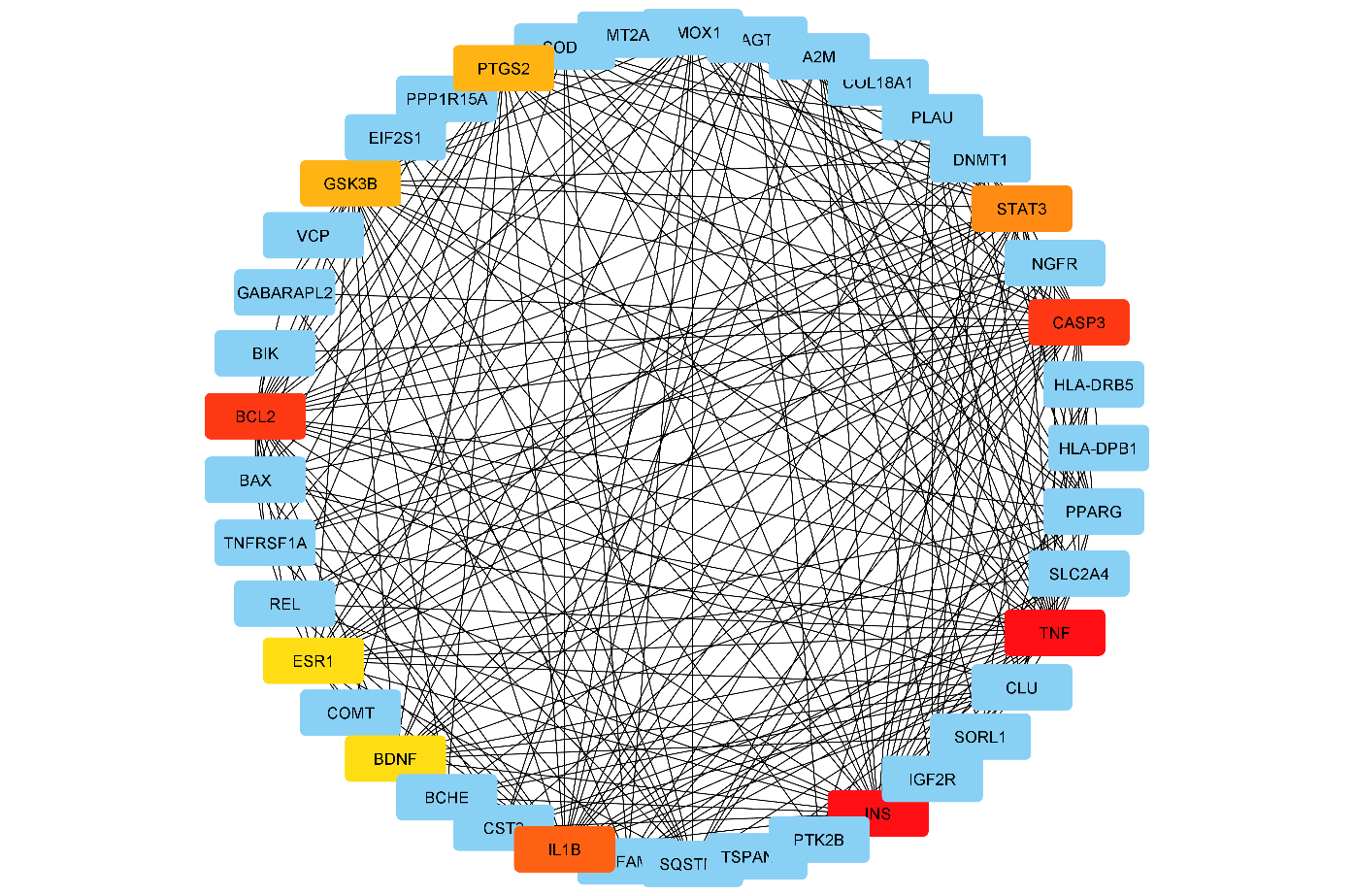 |
| **B** |
| 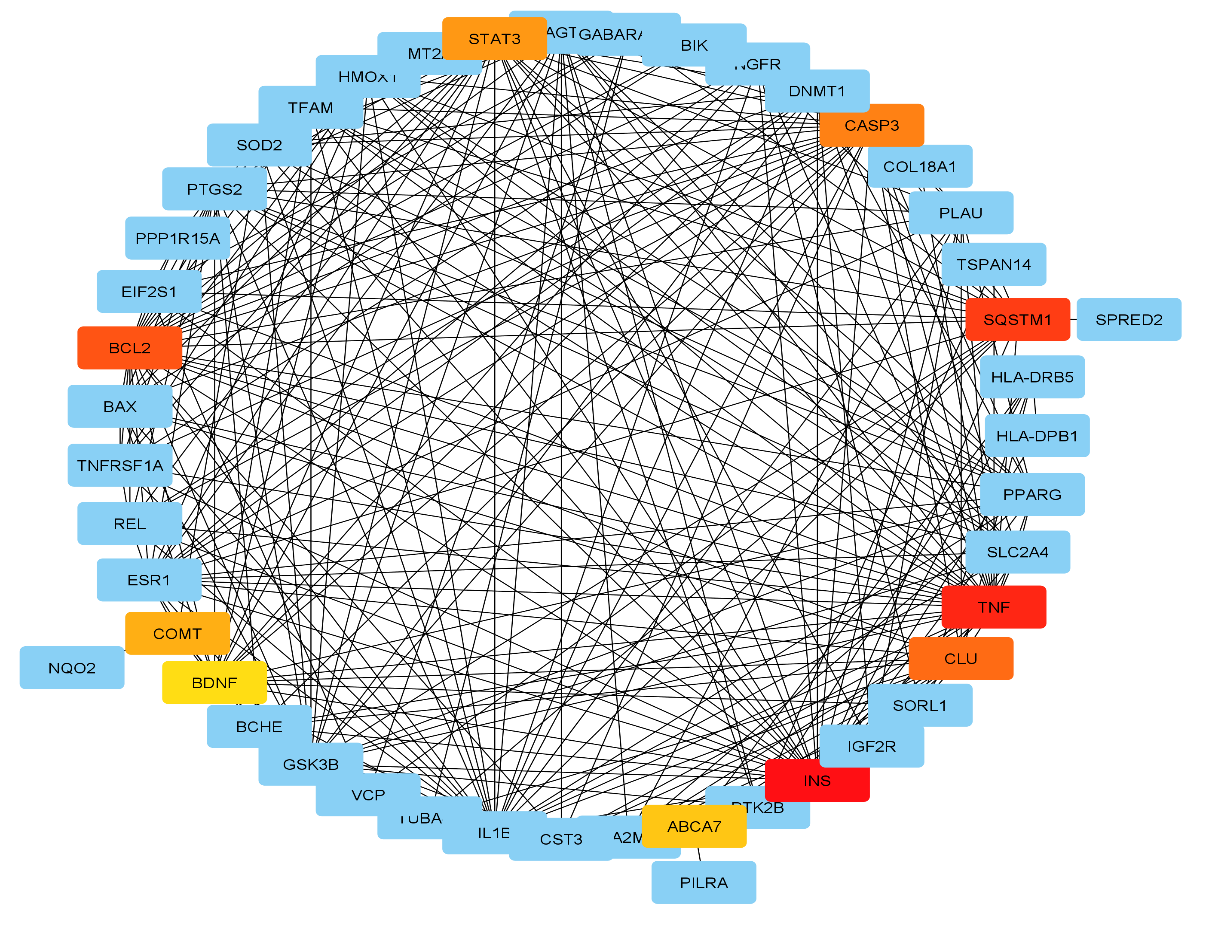 |
| **C** |
| 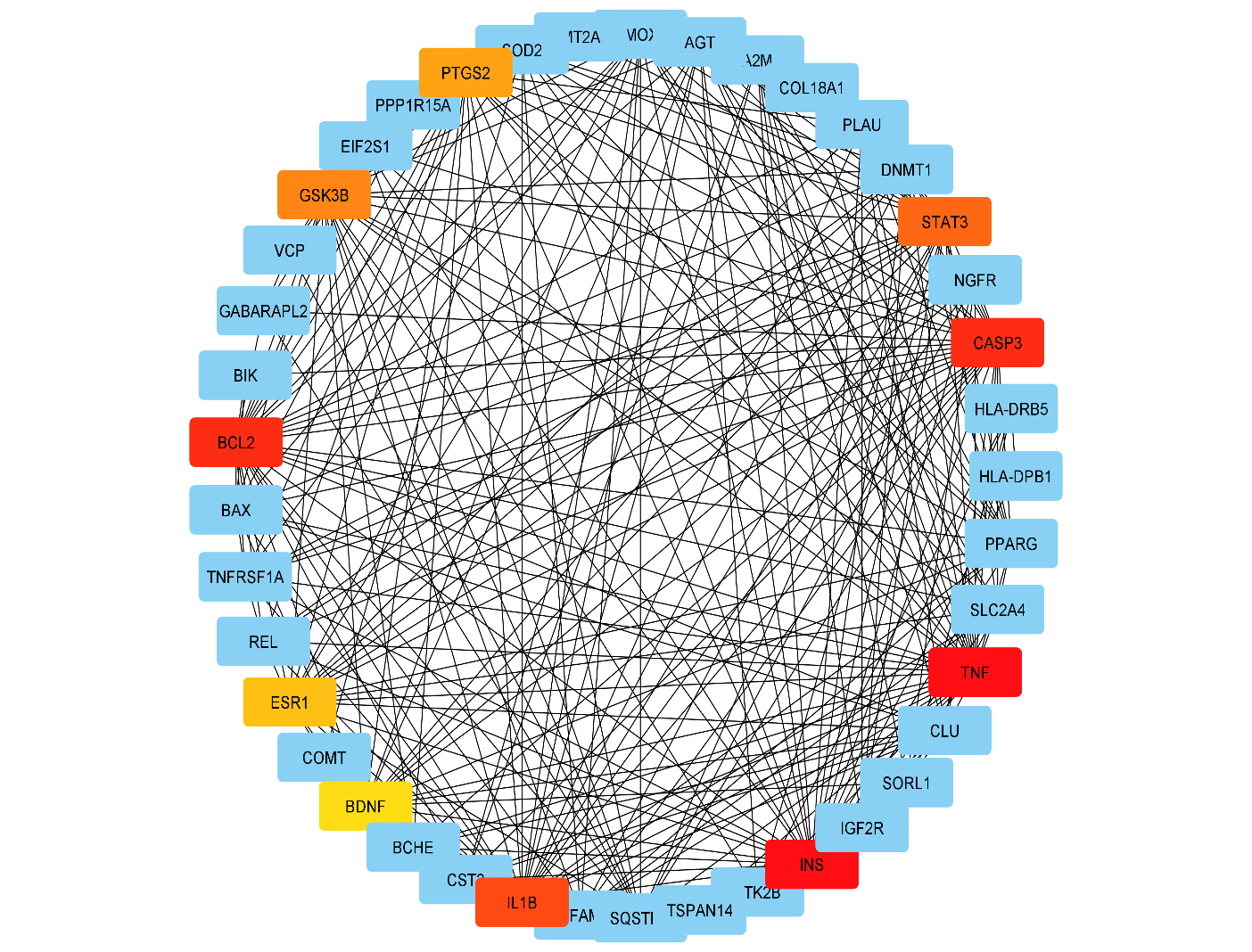 |

**Figure S1.** Network of targets implicated in the pathogenesis of Alzheimer’s disease and targeted by sulforaphane based on Degree, betweenness, and closeness (CytoHubba plug-in, Cytoscape software versions 3.9.1).

| **A** |
| --- |
| 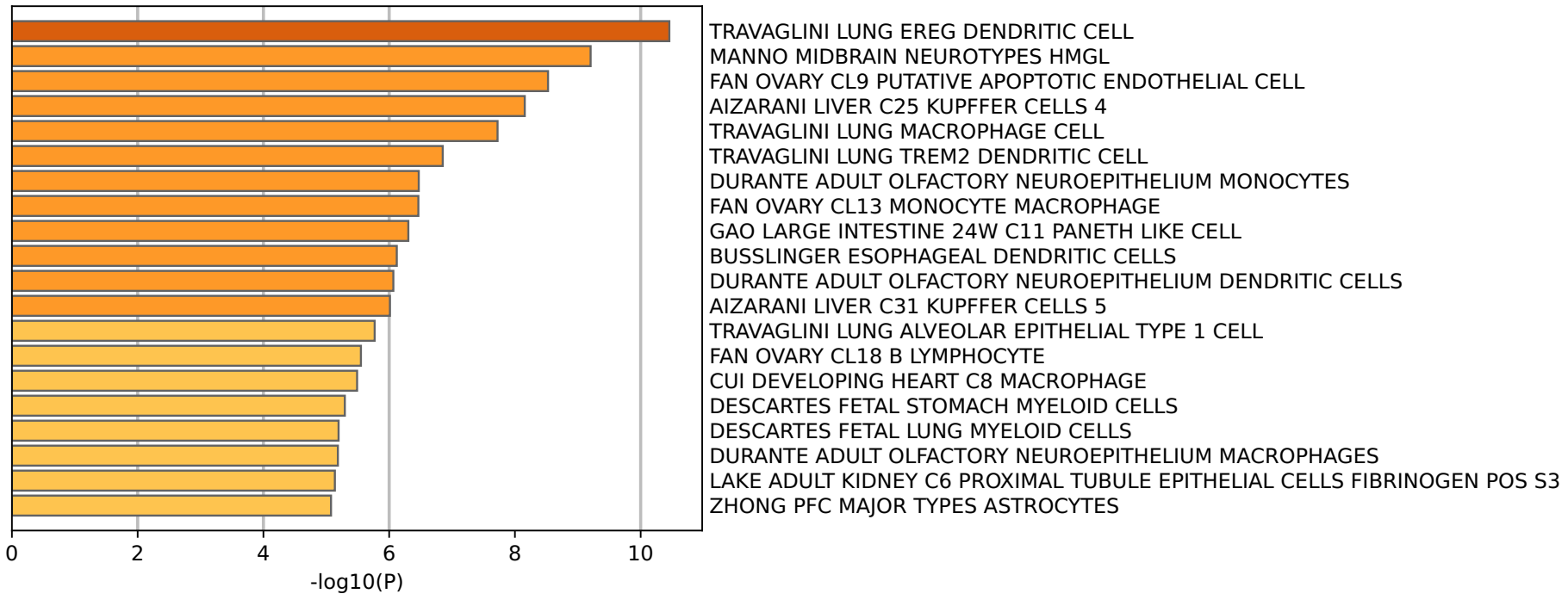 |
| **B** |
| 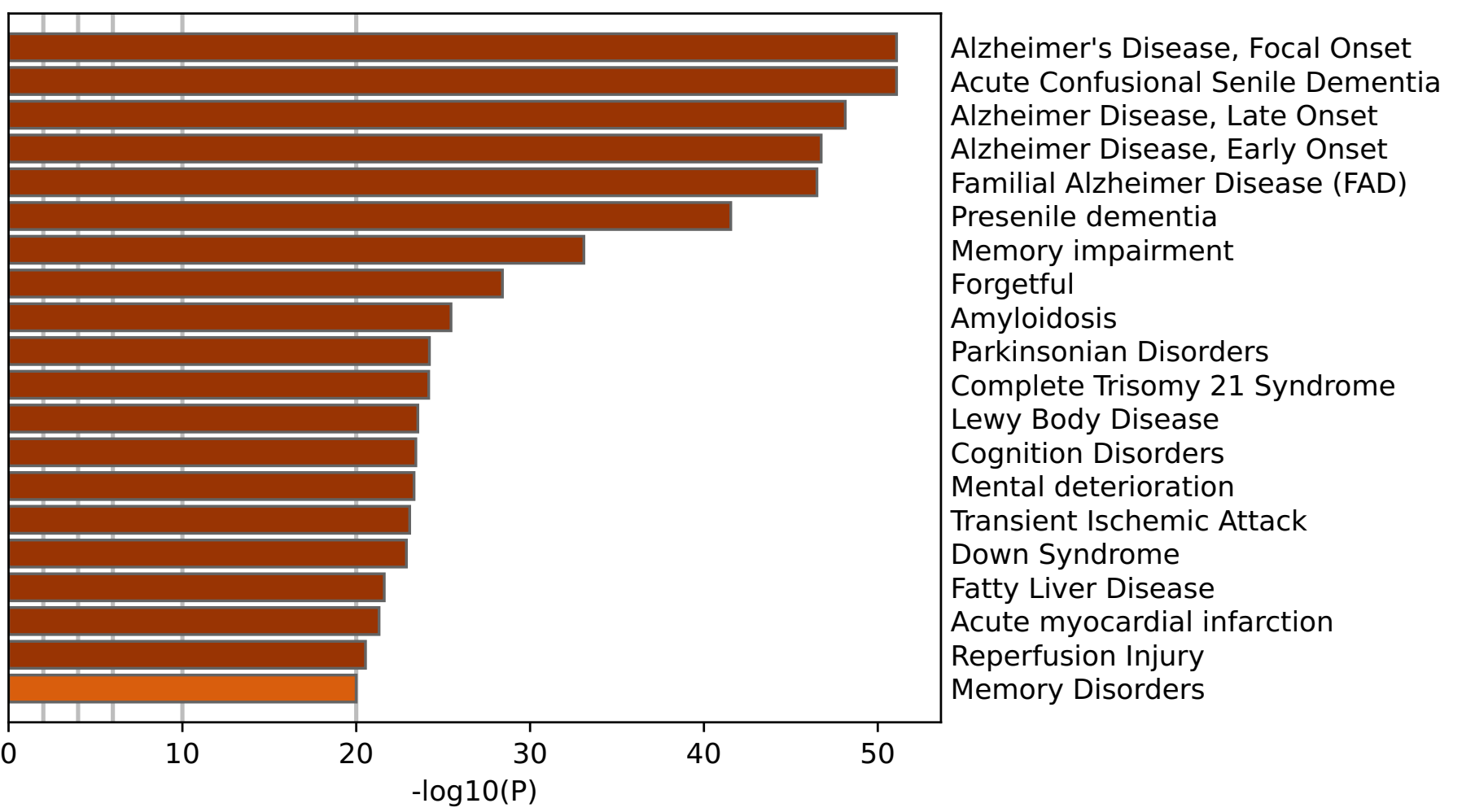 |
| **C** |
| 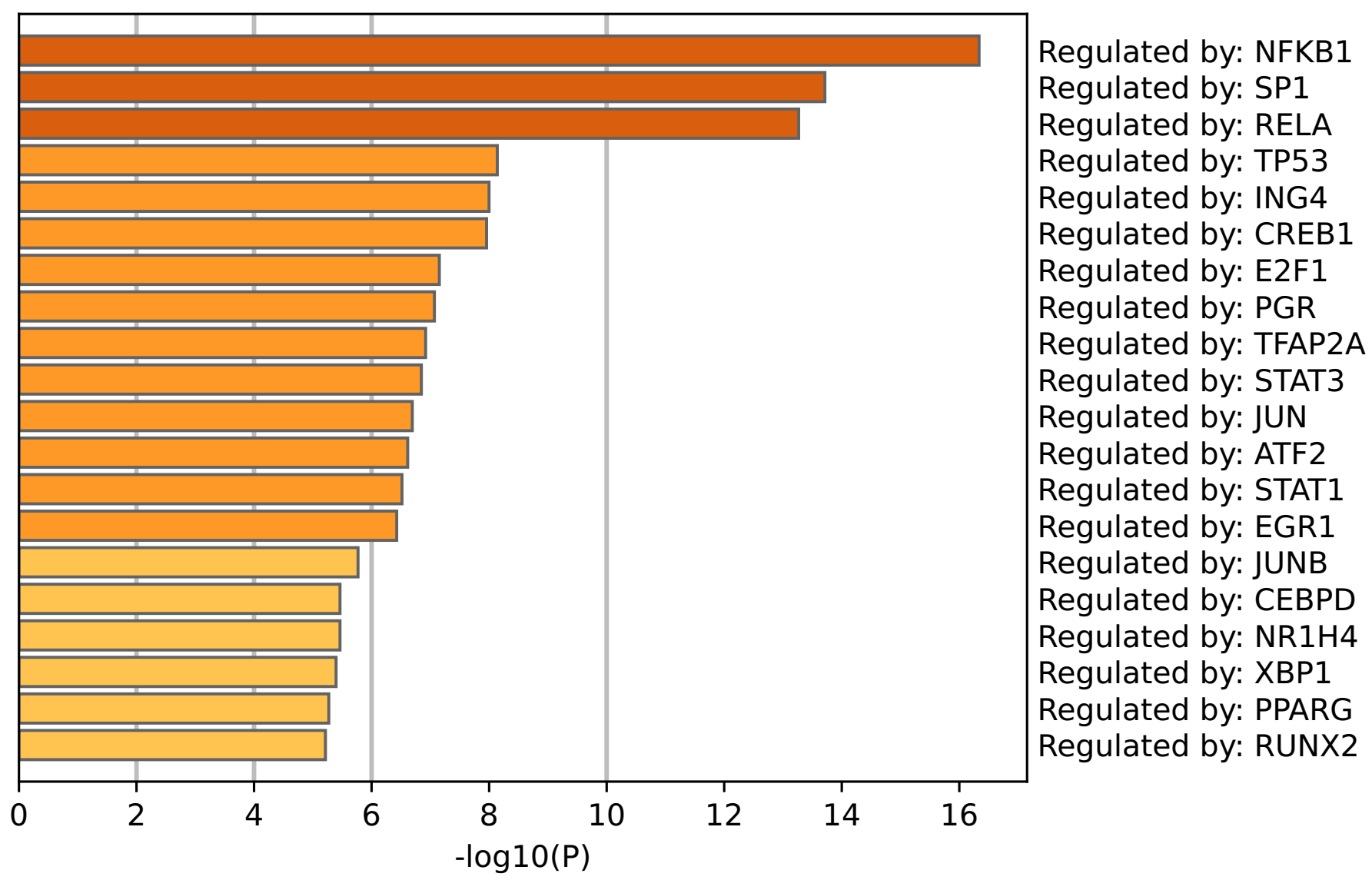 |

**Figure S2.** Specific cells, diseases, and transcription factors implicated in the pathogenesis of Alzheimer’s disease and targeted by sulforaphane (Metascape, https://metascape.org).

| **A** |  |  |
| --- | --- | --- |
| MicroRNA: | Site of interaction: | Total score: (Sorted by free energy) |
| hsa-miR-26b-5p | **site:1 -37.9 kcal/mol      site:26 -37.6 kcal/mol** **U** **miRNA 3'-UGGAUAGGACUUAAUGAACUU     UGGAUAGGACUUAAUGAACU** **\|\|\|\|\|\|\|\|\|\|\|\|\|\|\|\|\|\|\|\|\|     \|\|\|\|\|\|\|\|\|\|\|\|\|\|\|\|\|\|\|\|** **sponge 5'-ACCUAUCCUGAAUUACUUGAA     ACCUAUCCUGAAUUACUUGA** **AGAGG                    A** | Free energy of duplex: **-75.5** kcal/mol |

| 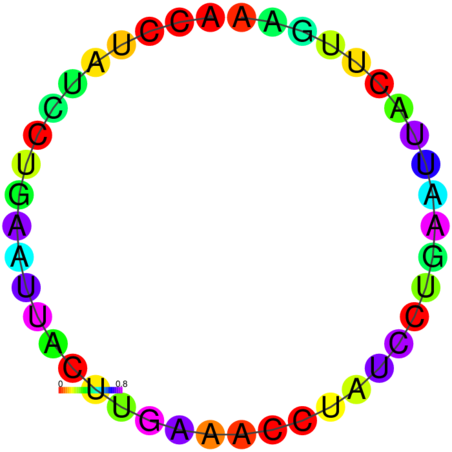 | 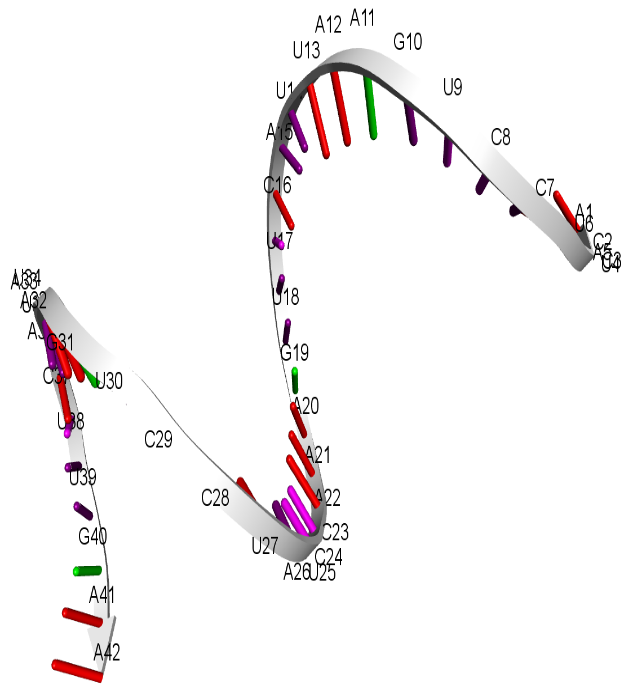 |
| --- | --- |

| **B** |  |  |
| --- | --- | --- |
| MicroRNA: | Site of interaction: | Total score: (Sorted by free energy) |
| hsa-miR-16-5p | **site:1 -42.8 kcal/mol       site:27 -42.2 kcal/mol**                                      **miRNA 3'-GCGGUUAUAAAUGCACGACGAU     GCGGUUAUAAAUGCACGACGAU** **\|\|\|\|\|\|\|\|\|\|\|\|\|\|\|\|\|\|\|\|\|\|     \|\|\|\|\|\|\|\|\|\|\|\|\|\|\|\|\|\|\|\|\|\|** **sponge 5'-CGCCAAUAUUUACGUGCUGCUA     CGCCAAUAUUUACGUGCUGCUA** **AGAGG** | Free energy of duplex: **-85** kcal/mol |

| 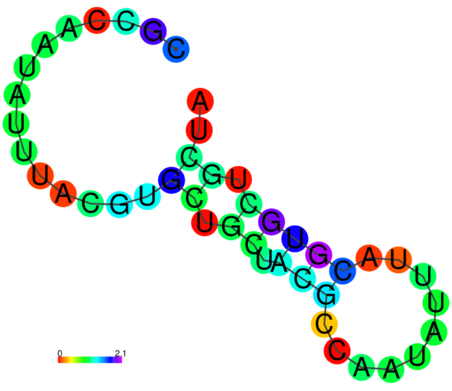 | 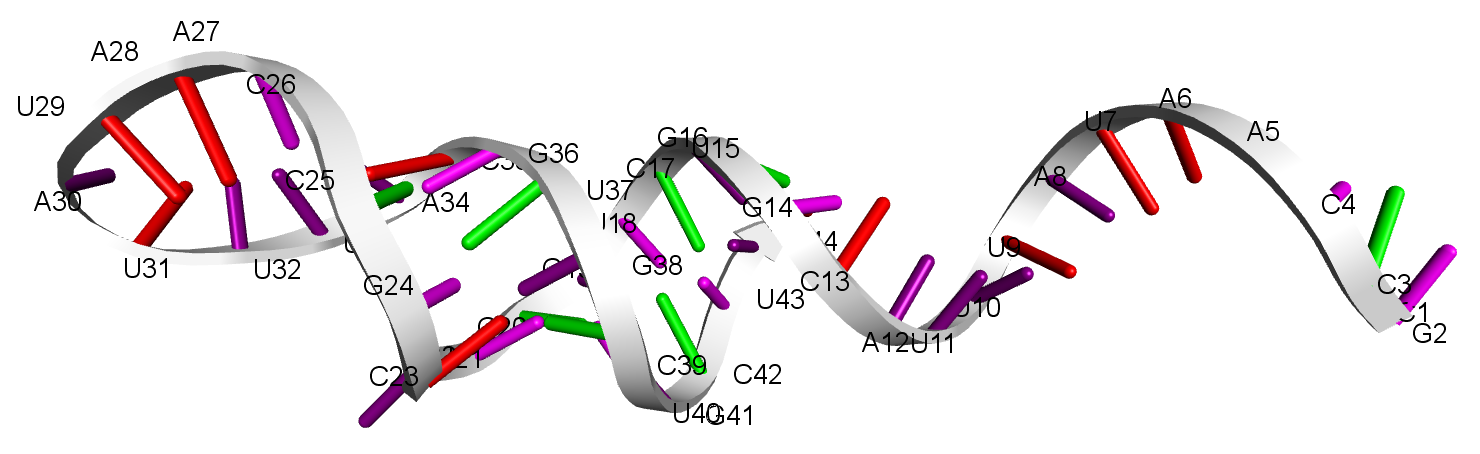 |
| --- | --- |

| **C** |  |  |
| --- | --- | --- |
| MicroRNA: | Site of interaction: | Total score: (Sorted by free energy) |
| hsa-miR-17-5p | **site:1 -46.3 kcal/mol        site:28 -45.4 kcal/mol**                                       **miRNA 3'-GAUGGACGUGACAUUCGUGAAAC     GAUGGACGUGACAUUCGUGAAAC** **\|\|\|\|\|\|\|\|\|\|\|\|\|\|\|\|\|\|\|\|\|\|\|     \|\|\|\|\|\|\|\|\|\|\|\|\|\|\|\|\|\|\|\|\|\|\|** **sponge 5'-CUACCUGCACUGUAAGCACUUUG     CUACCUGCACUGUAAGCACUUUG** **AGAGG** | Free energy of duplex: **-91.7** kcal/mol |

| 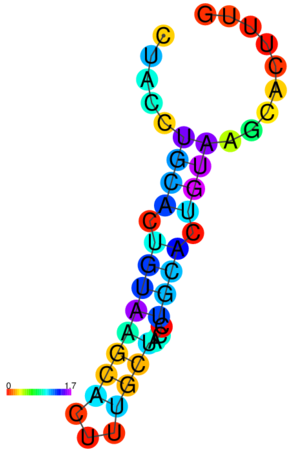 | 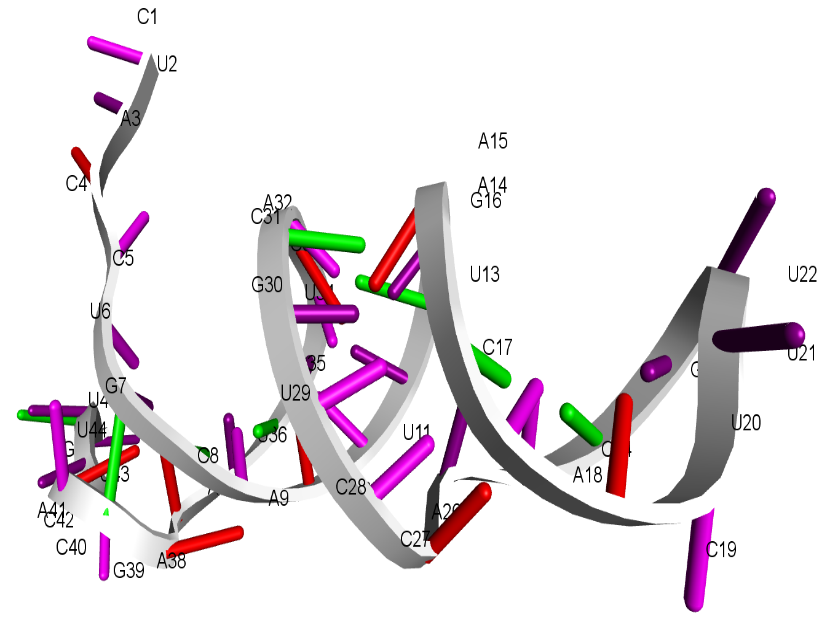 |
| --- | --- |

**Figure S3.** miRNA sponge structures for hsa-miR-17-5p, hsa-miR-16-5p, and hsa-miR-26b-5p (miRNAsong, https://www2.med.muni.cz/histology/miRNAsong/index.php)


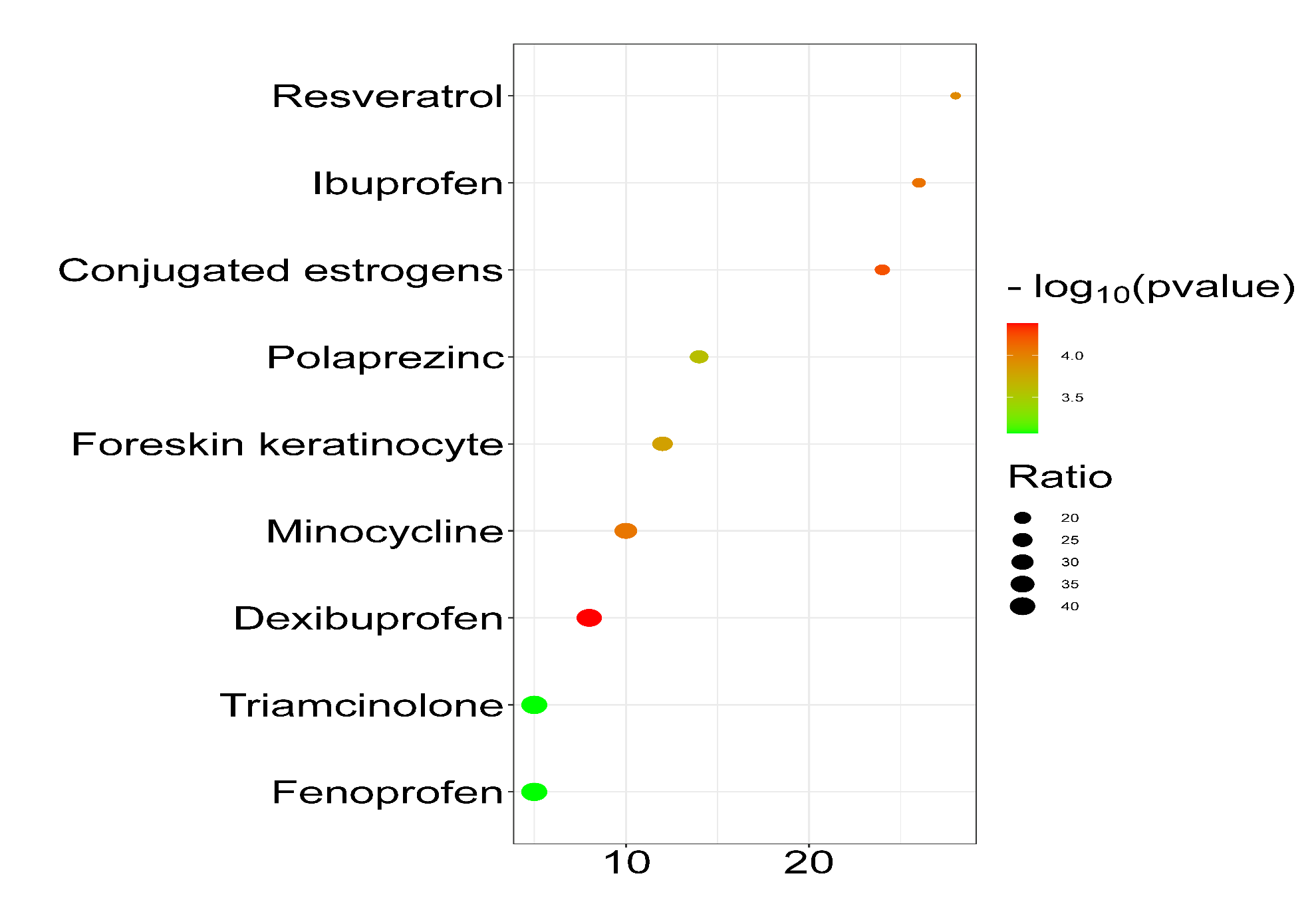


**Figure S4.** Drugs implicated in the pathogenesis of Alzheimer’s disease and targeted by sulforaphane (WebGestalt, http://www.webgestalt.org/).

**
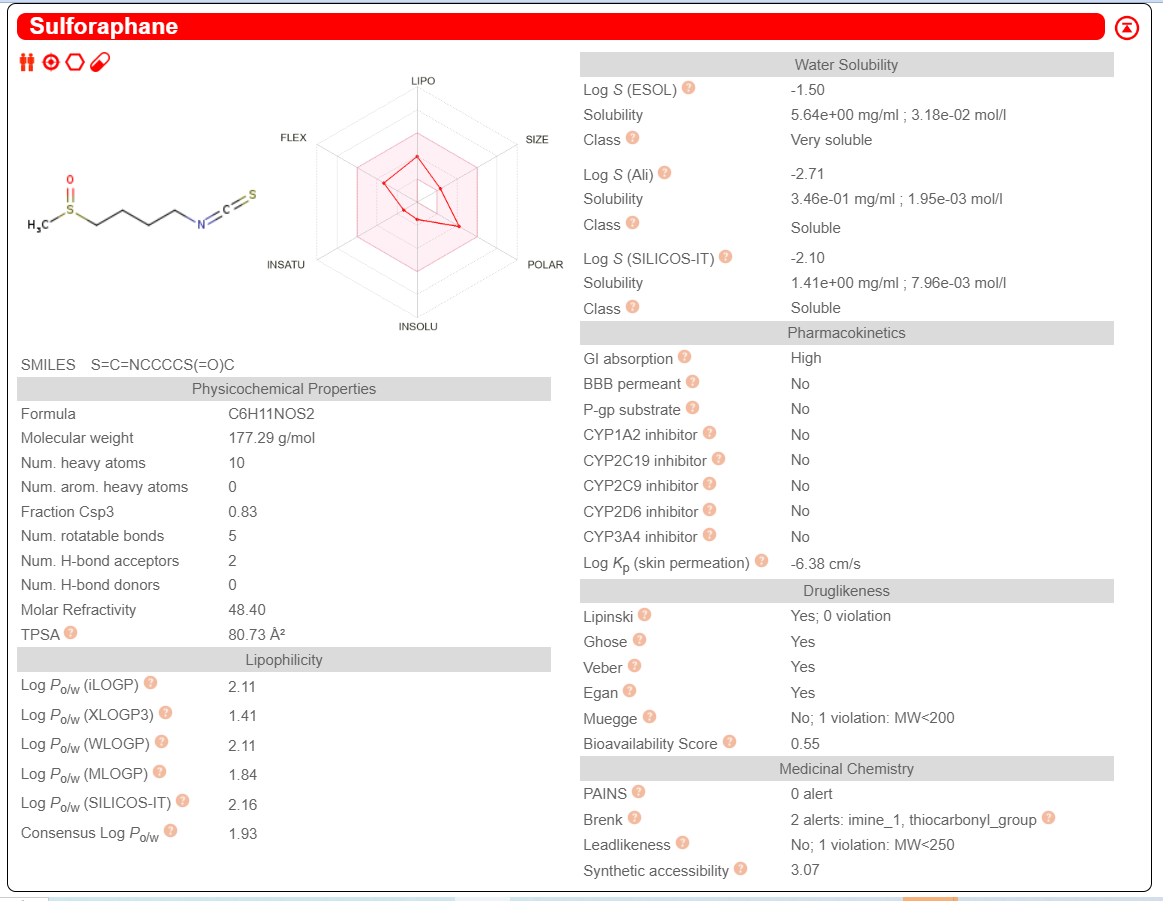
**

**Figure S5.** Physicochemical properties, pharmacokinetics, and druglikeness of sulforaphane (Swissadme, http://www.swissadme.ch/index.php).

**Table S1.** Forty-five targets implicated in the pathogenesis of Alzheimer’s disease and targeted by sulforaphane (CTD Database, http://CTD.mdibl.org).

| A2M | CLU | HMOX1 | PTK2B | TNF |
| --- | --- | --- | --- | --- |
| ABCA7 | COMT | IGF2R | REL | TSPAN14 |
| ABCC4 | CST3 | IL1B | SLC2A4 | TUBA4A |
| AGT | DHCR24 | INS | SLC51A | VCP |
| AKAP5 | DNMT1 | MT2 | SOD2 | VEGFA |
| BAX | EIF2S1 | NQO2 | SORL1 |  |
| BCHE | ESR1 | PILRA | SPRED2 |  |
| BCL2 | GSK3B | PLAU | SQSTM1 |  |
| BDNF | HLA-DPB1 | PPARG | STAT3 |  |
| CASP3 | HLA-DRB5 | PTGS2 | TFAM |  |

**Table S2.** Post-translational modification’s implicated in the pathogenesis of Alzheimer’s disease and targeted by sulforaphane (Uniprot, https://www.uniprot.org/).

| **Protein Name** | **Feature Type** | **Loc Start** | **Loc End** | **Description** | **Evidences** |
| --- | --- | --- | --- | --- | --- |
| TNF (P01375 · TNFA_HUMAN) | Chain | 1 | 233 | Intracellular domain 2 |  |
| TNF (P01375 · TNFA_HUMAN) | Chain | 50 | ? | C-domain 1 |  |
| TNF (P01375 · TNFA_HUMAN) | Chain | 52 | ? | C-domain 2 |  |
| TNF (P01375 · TNFA_HUMAN) | Chain | 77 | 233 | Tumor necrosis factor, soluble form1 Publication | PMID: 3856324 |
| TNF (P01375 · TNFA_HUMAN) | Disulfide bond | 145 | 177 | -- |  |
| ISN (P01308 · INS_HUMAN) | Signal | 1 | 24 | -- | PMID: 14426955 |
| ISN (P01308 · INS_HUMAN) | Peptide | 25 | 54 | Insulin B chain |  |
| ISN (P01308 · INS_HUMAN) | Disulfide bond | 31 | 96 | Interchain (between B and A chains) | PMID: 1433291, PMID: 25423173, PMID: 8421693 |
| ISN (P01308 · INS_HUMAN) | Disulfide bond | 43 | 109 | Interchain (between B and A chains) | PMID: 1433291, PMID: 25423173, PMID: 8421693 |
| ISN (P01308 · INS_HUMAN) | Propeptide | 57 | 87 | C peptide |  |
| ISN (P01308 · INS_HUMAN) | Peptide | 90 | 110 | Insulin A chain |  |
| ISN (P01308 · INS_HUMAN) | Disulfide bond | 95 | 100 | -- | PMID: 1433291, PMID: 25423173, PMID: 5101771, PMID: 8421693, PMID: 9235985 |
| BCL2 (P10415 · HUMAN) | Chain | 1 | 239 | Apoptosis regulator Bcl-2 |  |
| BCL2 (P10415 · HUMAN) | Modified residue | 69 | ? | Phosphothreonine; by MAPK8 | PMID: 18570871 |
| BCL2 (P10415 · HUMAN) | Modified residue (large scale data) | 69 | ? | Phosphothreonine | PMID: 31819260 |
| BCL2 (P10415 · HUMAN) | Modified residue | 70 | ? | phosphothreonine; by MAPK8 and PKC | PMID: 18570871 |
| BCL2 (P10415 · HUMAN) | Modified residue (large scale data) | 74 | ? | Phosphothreonine | PMID: 31819260 |
| BCL2 (P10415 · HUMAN) | Modified residue | 87 | ? | Phosphothreonine; by MAPK8 | PMID: 18570871 |
| BCL2 (P10415 · HUMAN) | Modified residue (large scale data) | 87 | ? | Phosphothreonine | PMID: 31819260 |

**Table S3.** Protein-protein interactions implicated in the pathogenesis of Alzheimer’s disease and targeted by sulforaphane (Metascape, https://metascape.org).

| **MCODE** | **GO** | **Description** | **Log10(P)** |
| --- | --- | --- | --- |
| MCODE_1 | GO:0001541 | Ovarian follicle development | -8.3 |
| MCODE_1 | GO:0008585 | Female gonad development | -7.5 |
| MCODE_1 | GO:0046545 | Development of primary female sexual characteristics | -7.4 |
| MCODE_2 | M36 | PID IL27 PATHWAY | -9.2 |
| MCODE_2 | M196 | PID IL23 PATHWAY | -8.8 |
| MCODE_2 | R-HSA-6783783 | Interleukin-10 signaling | -8.5 |
| MCODE_3 | WP2380 | Brain-derived neurotrophic factor (BDNF) signaling pathway | -7 |
| MCODE_3 | GO:0046777 | protein autophosphorylation | -6.6 |
| MCODE_3 | GO:0043087 | Regulation of GTPase activity | -5.8 |
| - | GO:0030162 | Regulation of proteolysis | -15.3 |
| - | GO:2001234 | Negative regulation of apoptotic signaling pathway | -15 |
| - | GO:2001233 | Regulation of apoptotic signaling pathway | -14.2 |

**Table S4.** ADMET properties for sulforaphane (admetlab 1.0, http://lmmd.ecust.edu.cn/admetsar1, and admetlab 2.0, https://admetmesh.scbdd.com/service/evaluation/index).

| **Model** | **Result** | **Probability** |
| --- | --- | --- |
| **Absorption** |  |  |
| Blood-Brain Barrier | BBB+ | 0.9705 |
| Human Intestinal Absorption | HIA+ | 0.8359 |
| Caco-2 Permeability | Caco2+ | 0.5096 |
| P-glycoprotein Substrate | Non-substrate | 0.6251 |
| P-glycoprotein Inhibitor | Non-inhibitor | 0.7887 |
|  | Non-inhibitor | 0.9957 |
| **Distribution** |  |  |
| Subcellular localization | Lysosome | 0.6263 |
| Distribution (VDss) | High | 1.076 |
| **Metabolism** |  |  |
| CYP450 2C9 Substrate | Non-substrate | 0.8211 |
| CYP450 2D6 Substrate | Non-substrate | 0.7566 |
| CYP450 3A4 Substrate | Non-substrate | 0.5858 |
| CYP450 1A2 Inhibitor | Non-inhibitor | 0.6705 |
| CYP450 2C9 Inhibitor | Non-inhibitor | 0.8369 |
| CYP450 2D6 Inhibitor | Non-inhibitor | 0.8893 |
| CYP450 2C19 Inhibitor | Non-inhibitor | 0.7584 |
| CYP450 3A4 Inhibitor | Non-inhibitor | 0.9586 |
| CYP Inhibitory Promiscuity | Low CYP Inhibitory Promiscuity | 0.9262 |
| **Excretion** |  |  |
| Clearance | Moderate | 7.700 |
| T1/2 | High | 0.855 |
| **Toxicity** |  |  |
| AMES Toxicity | Non AMES toxic | 0.5666 |
| Fish Toxicity | Low FHMT | 0.8208 |
| Tetrahymena Pyriformis Toxicity | Low TPT | 0.5664 |
| Biodegradation | Not ready biodegradable | 0.9251 |
| Rat Acute Toxicity | 2.3529 | LD50, mol/kg |
| Fish Toxicity | 2.4996 | pLC50, mg/L |
| Tetrahymena Pyriformis Toxicity | -0.0686 | pIGC50, ug/L |
